# Innovating health prevention models in detecting infectious disease outbreaks through social media data: an umbrella review of the evidence

**DOI:** 10.3389/fpubh.2024.1435724

**Published:** 2024-11-22

**Authors:** Monica Giancotti, Milena Lopreite, Marianna Mauro, Michelangelo Puliga

**Affiliations:** ^1^Department of Law, Economics and Social Sciences, Magna Graecia University, Catanzaro, Italy; ^2^Department of Economics, Statistics and Finance, University of Calabria, Cosenza, Italy; ^3^Department of Clinical and Experimental Medicine, Magna Graecia University, Catanzaro, Italy; ^4^Scuola Superiore Sant’Anna, Institute of Management, Pisa, Italy

**Keywords:** health policy, emerging infectious diseases, umbrella review, social media, surveillance

## Abstract

**Introduction and objective:**

The number of literature reviews examining the use of social media in detecting emerging infectious diseases has recently experienced an unprecedented growth. Yet, a higher-level integration of the evidence is still lacking. This study aimed to synthesize existing systematic literature reviews published on this topic, offering an overview that can help policymakers and public health authorities to select appropriate policies and guidelines.

**Methods:**

We conducted an umbrella review: a review of systematic reviews published between 2011 and 2023 following the PRISMA statement guidelines. The review protocol was registered in the PROSPERO database (CRD42021254568). As part of the search strategy, three database searches were conducted, specifically in PubMed, Web of Science, and Google Scholar. The quality of the included reviews was determined using A Measurement Tool to Assess Systematic Reviews 2.

**Results:**

Synthesis included 32 systematic reviews and 3,704 primary studies that investigated how the social media listening could improve the healthcare system’s efficiency in terms of a timely response to treat epidemic situations. Most of the included systematic reviews concluded showing positive outcomes when using social media data for infectious disease surveillance.

**Conclusion:**

Systematic reviews showed the important role of social media in predicting and detecting disease outbreaks, potentially reducing morbidity and mortality through swift public health action. The policy interventions strongly benefit from the continued use of online data in public health surveillance systems because they can help in recognizing important patterns for disease surveillance and significantly improve the disease prediction abilities of the traditional surveillance systems.

**Systematic Review Registration:**

http://www.crd.york.ac.uk/PROSPERO, identifier [CRD42021254568].

## 1 Introduction

In recent years, governmental responses to emerging infectious diseases (EIDs)—including severe acute respiratory syndrome (SARS) in 2003 (1, 2), H1N1 influenza in 2009, and the Ebola outbreak in 2014-2016—have proven insufficient, with health security measures often falling short.

As a result, the risks of epidemics were frequently underestimated, and funding for preventive interventions was inadequate. This under-preparation was evident during the early stages of the COVID-19 pandemic, with shortages of critical supplies like masks and respirators.

The COVID-19 pandemic underscored the necessity of developing early warning systems able to detect potential outbreaks through advanced data analysis. These systems are essential to prevent healthcare facilities from being overwhelmed in emergencies. One promising approach to addressing this challenge is the digitalization of health services. The use of social media (SM) platforms and digital tools by institutions during pandemics facilitates the rapid and widespread dissemination of information and the real-time tracking of disease outbreaks.

The emphasis is on the timeliness and immediacy required from the “first alert” health monitoring systems, and on effective “now casting” actions to counteract the rapidly developing of pre-pandemic events (3). Although the COVID-19 pandemic increased interest in health monitoring systems, digital epidemiology, which focuses on monitoring online and SM activities for public health purposes, has been explored since the early 2010s. Its primary goal is to capture health-related patterns and forecast disease outbreaks (3).

For instance, Al-Garadi et al. (3) conducted a systematic review in 2016, analyzing empirical studies from 2004 to 2015 on the use of SM data for infectious disease surveillance. The review highlighted the potential of platforms like Twitter and Facebook to track diseases such as influenza, Ebola, and Zika. The findings showed that SM data could predict pandemic trends earlier than official sources, despite challenges related to data limitations, algorithms, and privacy.

However, several limitations remain. SM platforms can be biased toward specific countries or regions, i.e., Twitter or Facebook do not represent countries such as China and Russia, or India. Instead, other SM will work only in given countries (i.e., Baidoo in China). Moreover, tracking and monitoring contacts, critical in disease prevention, can be hindered by privacy restrictions. Social interaction graphs based on SM data are often incomplete, limiting the ability to track real contact networks with the risk of losing important information for disease tracking. Additionally, misinformation, fake news, and echo chambers discussing diseases and outbreaks can distort health signals in SM discussions and posts. Researchers need to develop methods to mitigate these limitations by statistically correcting population biases and creating frameworks to counteract misinformation. This could involve boosting official information posts and highlighting credible sources with the support of SM service providers.

Despite these challenges, previous studies have demonstrated the potential of SM listening to reduce both morbidity and mortality by enabling swifter public health responses. According to the According to the World Health Organization (WHO), 51% of outbreak information related to EIDs between 2001 and 2011 was initially sourced from SM platforms (4).

Facebook, Twitter, Instagram, and Reddit are among the most widely used SM platforms globally (2–5). Users spend significant amounts of time online, sharing vast amounts of information. These data provide valuable insights into community health, offering a low-cost means to observe large populations (4, 6) and study health conditions at scale (3, 7). SM data can also help geo-localize potential chains of contagion (8). For example, Dong et al. (5) used spatial–temporal analysis of SM data in 2016 to track geographical information related to disease outbreaks such as flu and hay fever, demonstrating that SM data can predict disease spread.

As such, SM serves as a complementary tool to traditional surveillance systems, capable of detecting latent pre-pandemic phenomena and potentially anticipating successive waves of ongoing epidemics.

Recognizing this potential, the European Center for Disease Prevention and Control (ECDC) launched “Epitweetr” in October 2020, an open-source software for processing Twitter data to monitor epidemic events.

Further evidence supporting the importance of digital tools in detecting epidemic signals comes from a study by Kogan et al. (9). This research demonstrated how platforms like Google searches and Twitter can capture early signals of potential public health threats (10).

In this framework, the rapid increase in empirical studies into the use of SM in detecting EID has been paralleled with a comparable increase in systematic literature reviews on this topic.

However, no comprehensive summary has yet been provided to consolidate these findings and offer general conclusions that could guide healthcare systems in understanding the advantages and limitations of using SM data for infectious disease surveillance. To address this gap, we conducted an umbrella review—a synthesis of existing systematic literature reviews (11). This study compiles the findings of previous systematic reviews on this topic, offering policymakers and public health authorities an overview to inform policy decisions and guidelines. The structure of the paper is as follows: the next section describes the methods used in the review, followed by the results in Section 3. Discussions, conclusions, and policy implications are presented in Section 4.

## 2 Materials and methods

The review protocol was registered in the Prospective Register of Systematic Reviews (PROSPERO ID: CRD42021254568). The Preferred Reporting Items for Overviews of Reviews (PRIOR) (12) and the methodological considerations when using existing systematic reviews (13) guided the method and the reporting of findings (The complete PRISMA Checklist is provided in Supplementary Table 1).

### 2.1 Research question/objective

Our investigation is driven by the following research question: *What we know from the systematic reviews on the role of SM as early warnings tools of an epidemic outbreak?*

Although there were no restrictions on specific SM platforms, this study focused on major platforms like Facebook, Sina Weibo, Instagram, Reddit, YouTube, and Twitter. We considered those diseases that for their potential epidemic exhibited the greatest public health risk: COVID-19, Crimean-Congo hemorrhagic fever, Ebola virus disease, Marburg virus disease, Lassa fever, Middle East respiratory syndrome coronavirus (MERS-CoV), SARS, Nipah and henipaviral diseases, Rift Valley fever, Zika (14). The study also examined the H1N1 influenza. While it was not declared a pandemic until 2010, it closely resembles the COVID-19 emergency in terms of epidemiology, clinical behavior, and the need for robust health policy interventions.

### 2.2 Search strategy and eligibility criteria

We developed a search strategy using similar reviews (15–17). From March 2023 to 1 October 2023, we systematically searched studies using Web of Science and PubMed databases. We chose these databases for their extensive journal coverage in health sciences. Keywords for the query were manually selected from relevant studies for a comprehensive list of cited papers and publishing trends. Specifically, the search strategy combined terms for individual EIDs with terms of health and SM, and terms relative to a range of systematic review types (Table 1).

**Table 1:** Databases and search strategy.

Database	Search strategy
*PubMed* (http://www.ncbi.nlm.nih.gov/pubmed/); *Web of science* (https://apps.webofknowledge.com); *Google scholar* (https://scholar.google.com/)	(Infectious disease* OR *COVID-19* OR *Ebola* OR *MERS* OR *SARS* OR *Zika* OR *H1N1* OR *Pandemic*) AND (Health^*^ OR early warning* OR Outbreak* OR *surveillance*) AND (*social media* OR *social network* OR *Facebook* OR *Youtube* OR *Facebook* OR *Instagram* OR *Sina Weibo* OR *Reddit* OR *Twitter*) AND (review OR systematic review – in title)

The search of the database has been extended to the title, keywords, and abstracts (topics range). Only the keywords “review” or “systematic review” were restricted to the title. Only studies published in English were included, due to resource limitations for translating non-English publications. Gray literature was identified using the Google Scholar search engine, with results examined up to the 20th page.

Two investigators independently reviewed potentially eligible articles. The inclusion criteria focused on systematic reviews that investigated: (a) the use of SM for early detection of infectious disease outbreaks, (b) the role of SM as a source of information for improving healthcare system efficiency, and (c) the contribution of SM in tracking and monitoring infectious disease outbreaks and preparing timely interventions. Excluded studies were those that: (a) were not systematic reviews, (b) did not focus on SM’s role in detecting or predicting EIDs, or (c) solely described early warning systems without involving SM. Based on these criteria, two authors screened the complete list of titles generated by the search procedure, creating a preliminary classification list. The articles went through abstract review and the full text was obtained for potentially relevant ones.

Then, two other authors searched the references of the selected articles to identify further relevant studies. Discrepancies in paper inclusion were resolved through discussions until a consensus was reached.

### 2.3 Data extraction

Data extraction was performed independently by all authors, with the selected papers divided equally among them. Data sheets were used to collect data on the following elements: reference, title, journal, publication year, number of citations, declaration of conflict of interest and funding (items included for quality assessment of systematic reviews), aim/research questions, databases and dates, type of EIDs, type of social network, total studies included, key findings/recommendations on the potential utility of SM in predicting infectious diseases outbreaks, and their potential limitations (Table 2). Missing data were filled in, when possible, by mail correspondence with the study authors. Results were presented narratively. A narrative synthesis is often employed in systematic reviews when a meta-analysis is impractical due to the diversity of studies included, such as variations in methods, populations, interventions, or outcomes In addition, narrative synthesis offers flexibility in recognizing and discussing shared themes, patterns, and trends within the studies, resulting in a more comprehensive grasp of the literature that may not be apparent through quantitative analysis alone (18, 19).

**Table 2:** Characteristics and summary of results of included systematic reviews.

N	Reference	Title	Number of citations (Source: Google scholar; 3 November 2023)	Databases and dates of search undertaken by the review	Total texts included	Type of EIDs included	Specific SNs cited in the review	Key findings	Limitations in using social network
1	Guy et al. 2012 (47)	Social media: A systematic review to understand the evidence and application in infodemiology.	22	EMBASE and PubMed: from 1999 to 2011	12	ILI, H1N1 Influenza	Twitter, Facebook	Studies included show the use of open-source micro-blogging sites to inform influenza-like-illness monitoring.	Data extracted from SM can be difficult to classify and interpret. Collected data may not be representative of the entire population. Not all profile accounts on networking sites contain geographic information; visible geographic information cannot be verified for accuracy.
2	Moorhead et al. 2013 (23)	A New Dimension of Health Care: Systematic Review of the Uses, Benefits, and Limitations of Social Media for Health Communication	2,538	CSA Illumina, Cochrane Library, Communication Abstracts, EBSCO Host CINAHL, ISI Web of Knowledge, Web of Science, OvidSP Embase, OvidSP MEDLINE, OVIDSP PsycINFO, and PubMeb Central: from January 2002 to February 2012	98	Unrestricted	Unrestricted (particularly cited Facebook, Twitter, YouTube, Blogs, MySpace)	In public health surveillance, SM can contribute to monitor public response to health issues, tracking and monitoring disease outbreak.	The main recurring limitations of SM are quality concerns and the lack of reliability of the health information.
3	Bernardo et al. 2013 (24)	Scoping Review on Search Queries and Social Media for Disease Surveillance: A Chronology of Innovation	197	SciVerse Scopus: from 2002 to 2011	51	Influenza, Foodborne/Gastroenteritis, Dengue fever, HIV	Twitter, Google, Facebook, Yahoo, Wiki	The reviewed literature highlighted accuracy, speed, and cost performance that was comparable to existing disease surveillance systems and recommended the use of SM programs to support those systems. International organizations could consider SM in a serious light, particularly as a means of engagement rather than just disseminating information.	While their accuracy, speed, and cost compare favorably with existing surveillance systems, the primary challenge is to refine the data signal by reducing surrounding noise.
4	Luan and Law 2014 (34)	Web GIS-Based Public Health Surveillance Systems: A Systematic Review	37	Geobase and PubMed: from 1 January 2000 and 31 March 2013	58	Unrestricted	Twitter	Even if SM cannot confirm an outbreak, it can contribute to an investigation and for timely collection of geo-referenced health data offering the possibility of large-scale public health surveillance.	SM have a limited ability to identify precise locations of areas of interest (e.g., disease clusters) and their corresponding characteristics.
5	Carrol et al. 2014 (35)	Visualization and analytics tools for infectious disease epidemiology: A systematic review	275	National Library of Medicine’s MEDLINE through PubMed, Cochrane Library, New York Academy of Medicine’s Gray Literature, Web of Science, and IEEE Digital Library: from January 1, 1980 to June 30, 2013	88	Unrestricted	Unrestricted	Social networks analyses can contribute to track the spread of infectious diseases, potential outbreaks, and prepare adequate interventions. It is especially useful in identifying the index or source case and predicting which individuals are more likely to become infected and further infect others.	Efforts to map users’ queries of common data types to meaningful visualizations have raised concerns regarding the potential for misinterpretation and cognitive overload due to the complexity of infectious disease data. Other problems are limitations of user knowledge and organizational capacity to implement specific tool for social networks analysis, as well as generation of accurate and easy-to-understand visualizations.
6	Velasco et al. 2014 (32)	Social Media and Internet-Based Data in Global Systems for Public Health Surveillance: A Systematic Review	279	PubMed, Scopus, and Scirus: from 1990 to 2011	32	Unresticted	Unrestricted	Literature does indicate that event-based surveillance could improve official surveillance activities.	Health authorities who intend to use content from SM and other Internet data also need to consider protection and privacy, such as legal and ethical implications related to using Internet and SM data for public health surveillance.
7	Charles-Smith et al. 2015 (25)	Using Social Media for Actionable Disease Surveillance and Outbreak Management: A Systematic Literature Review	335	PubMed, Embase, Scopus, and Ichushi-Web: from 2000 to February 2013	60	H1N1 Influenza, ILI, Dengue fever, Cholera, Campylo-bacteriosis outbreak, HIV, *Escherichia coli*	Facebook, MySpace, Twitter, Blogs, Discussion forums	SM is shown to be effective in improving public health and may be effective at disease surveillance. Public health should integrate SM analytics into disease surveillance and outbreak management practice. The use of SM data could provide real-time surveillance of health issues, speed up outbreak management, and identify target populations necessary to support and improve public health and intervention outcomes.	An ethical framework for the integration of SM into public health surveillance systems is missing.
8	Choi et al. 2016 (26)	Web-based infectious disease surveillance systems and public health perspectives: a systematic review	141	PubMed, Web of Science, and Embase databases: from 2000 to 2015	60	Unrestricted	Unrestricted	Web-based infectious disease surveillance systems exhibit clear strengths, as compared to traditional surveillance systems. The major strengths of the newly emerging surveillance systems are that they are intuitive, adaptable, low-cost, and operated in real-time, all of which are necessary features of an effective public health tool.	The most apparent potential challenges of the web-based systems are those of inaccurate interpretation and prediction of health status, and privacy issues, based on an individual’s internet activity.
9	Sinnenberg 2017 (44)	Twitter as a Tool for Health Research: a Systematic Review	660	PubMed, Embase, Web of Science, Google Scholar, and CINAHL: from 2010 to 2015	137	Influenza, Ebola virus	Twitter	Twitter is a valid tool for disease surveillance in health research.	Policies regarding privacy and consent of the users producing the messages have yet to be universally defined.
10	Al-Garadi et al. 2016 (3)	Using online social networks to track a pandemic: A systematic review	205	PUBMED, IEEExplore, ACM Digital Library, Google Scholar, and Web of Science: from 2004 to 2015	20	Seasonal flu, H1N1 Influenza, HIV, Influenza, ILI, Ebola virus, Zika virus	Facebook, Twitter, Myspace, YouTube, Linkedln, Google+, Friendster	Online social network (OSN) data contain significant information that can be used to track a pandemic.	The limitation in using OSN to track pandemic is in collecting representative data with sufficient population coverage. This challenge is related to the characteristics of OSN data. The data are dynamic, large-sized, and unstructured, thus requiring advanced algorithms and computational linguistics. In summary, OSN-based surveillance system requires comprehensive adoption, enhanced geographical identification system, and advanced algorithms and computational linguistics to eliminate its limitations and challenges. OSN is probably to never replace traditional surveillance, but it can offer complementary data that can work best when integrated with traditional data.
11	Fung et al. 2016 (38)	Ebola virus disease and social media: A systematic review	117	Web of Science, PubMed, and EBSCOhost: from 2013 to November 2015	12	Ebola virus	Twitter, Facebook, YouTube, Instagram, Flickr	Studies suggest that SM can improve public health communication surveillance.	Manual categorization of SM contents remains relevant to public health because high-level summaries of contents (ie, themes) still require human interpretation. There is a need to translate research development into public health routine practice.
12	Phillips et al. 2017 (39)	Using Social Media To Predict the Future: A Systematic Literature Review	83	IEEE, ACM, other sources: from 2010 to October 2016	106	H1N1 Influenza, ILI, Dengue fever	Unrestricted	Success in predicting epidemiological outbreaks using SM data was reported in various studies.	SM forecasting is limited by data biases, noisy data, lack of generalizable results, a lack of domain-specific theory, and underlying complexity in many prediction tasks.
13	Eckert et al. 2018 (37)	Health-Related Disaster Communication and Social Media: Mixed-Method Systematic Review	63	Web of Science; Google Scholar, PubMed/Medline-National Library of Medicine (NLM); Cumulative Index of Nursing and Allied Health Literature (CINAHL); CINAHL Complete; Communication and Mass Media Complete (CMMC); PsychInfo; and the website of the World Health Organization: from 2003 to 2016	79	Ebola virus, Avian influenza, H1N1 Influenza	Unrestricted (particularly cited Twitter, Facebook, Sina Weibo, WhatsApp, Flickr, SMS, blogs, YouTube)	SM, especially Twitter and Facebook, should be used by global, regional, and local government agencies, first responders, healthcare practitioners, and the public to monitor public reactions during a disaster; to address the public and to provide accurate, timely, and transparently source information.	SM are still tools that have not become routine practices in many governmental agencies regarding public health in the countries studied. Obstacles still include the reluctance to learn new ways to communicate, the lack of additional staff to handle the increase in information exchange needs via SM, and missing universal guidelines on best practices of SM in daily operations of public health officials and especially during public health crises events.
14	O’Shea 2017 (31)	Digital disease detection: A systematic review of event-based internet biosurveillance systems	79	PubMed, Scopus, and Google Scholar: from May 2011 to July 2015	99	Unrestricted	Unrestricted	The review emphasizes the importance of using both formal and informal sources, such as SM, for timely and accurate infectious disease outbreak surveillance.	Regulations are needed in SM as a data source, and all online data sources, to ensure good governance of the data and that individuals’ privacy is not violated.
15	Gianfredi et al. 2018 (22)	Harnessing Big Data for communicable tropical and Subtropical Disorders: implications From a Systematic Review of the Literature	38	PubMed/MEDLINE, Scopus, ISI/Web of Science: from 2014 to 2017	47	Zika virus, Ebola virus, Chikungunya, West Nile virus, Dengue fever, Mayaro virus	Google trends, Twitter, Facebook, YouTube, Instagram, Pinterest	Most of included studies found a strong correlation between SM posts and epidemiological cases. Novel data streams appear to be promising tools for predicting the spread of infectious agents, and, as such, can potentially aid and inform early decision support for when and how to employ public health interventions within a certain community.	In some cases, it was found that SM tweets disseminated spreading of conspiracy theories, pseudo-scientific claims and misleading information, particularly during the first weeks of pandemic.
16	Hagg et al. 2018 (40)	The emerging use of social media for health-related purposes in low and middle-income countries: A scoping review	127	The Cumulative Index to Nursing and Allied Health Literature (CINAHL), Medline/PubMed, Web of Science, CAB Direct (CAB Abstracts and Global Health), Compendex Engineering Village, and Compendex Engineering Village 2, Google Scholar: from 2010 to 2017	40	Ebola virus, Zika virus, MERS, H7N9, Dengue fever	Unrestricted (particularly cited Twitter, YouTube, Sina Weibo, Baidu, TB&Me blog, MoBuzz social network site, WhatsApp, Viber, Google Trends)	SM can facilitate disease surveillance.	Misinformation or poorly communicated information can contribute to negative health behaviors and adverse health outcomes among consumers, as well as hysteria and chaos.
17	De Araujo et al. 2019 (27)	Social networks applied to Zika and H1N1 epidemics: a systematic review	10	Science Direct; Springer; Scielo; IEEE Xplorer	20	Zika virus, H1N1 Influenza	Unrestricted (particularly cited Facebook, Twitter)	Collaboration trough social networks generates information that allows the monitoring and control of epidemics.	Problems related to objectivity, dynamism, rumor and other aspects related to the quality of information could be diminish or at least improved.
18	Golinelli et al. 2020 (43)	Adoption of Digital Technologies in Health Care During the COVID-19 Pandemic: Systematic Review of Early Scientific Literature	353	MEDLINE and medRxiv: from January 1 to April 30, 2020	124	COVID-19	Unrestricted	SM can be useful for COVID-19 diagnosis as well as for implementing prevention and surveillance measures.	Despite social networks have already proven their effectiveness for surveillance, problems related to privacy remain.
19	Corsi et al. 2021 (46)	Big data analytics as a tool for fighting pandemics: a systematic review of literature	66	Web of Science and Scopus: no time restriction	45	Influenza, Ebola virus, Dengue fever, HIV, SARS-CoV, Zika virus, COVID-19, MERS, Chikungunya, Yellow fever, Chagas	Twitter, Facebook, Instagram, Pinterest, Sina Weibo, Sina Micro, Personal blogs, Naver	Many sources of data used in cases of previous epidemics and pandemics come from SM. SM are means of extracting the opinion of society in real-time, in addition to allowing the capture of geographic location information. Also, SM are tools contribute to explore in changing people’s behavior during disease outbreaks.	It is difficult to extract data from SM, given the heterogeneous characteristics, unstructured data, and dynamic change.
20	Gupta and Katarya 2020 (30)	Social media based surveillance systems for healthcare using machine learning: A systematic review	121	ACM Portal, IEEE Xplore, Science Direct, PubMed, Google Scholar: from 2010 to 2018	26	Ebola virus, Zika virus, ILI, Dengue fever, H1N1, MERS, Foodborne, Avian Influenza	Twitter, facebook, Instagram, Crowdsourcing, Other microblogs	In comparison to traditional surveillance systems, SM based surveillance systems show superiority.	The data collected from SM sites may contain irrelevant data. Much information can be a reflection of panic and not the real incidences of a disease outbreak. There is discussion about ethical concerns while retrieving data from SM, including the privacy of the datasets collected using SM for health purposes.
21	Barros et al. 2020 (28)	The Application of Internet-Based Sources for Public Health Surveillance (Infoveillance): Systematic Review	67	Europe PubMed Central, Institute of Electrical and Electronics Engineers Xplore Digital Library, Association for Computing Machinery Digital Library, SpringerLink, EBSCO Host, PubMed, Scopus, and Web of Science: from 2012 to 2018	162	Dengue fever, H1N1, Bird flu, ILI	Twitter, Facebook, Instagram, Weibo, YouTube, Daum	When dealing with outbreak detection, an early and fast response is essential. Traditional surveillance is slower to transmit information across its different channels; therefore, internet-based sources complement the traditional mechanism when dealing with outbreaks. Interned-based sources, which include SM, contribute to detect the first evidence of an outbreak. With the evidence provided by these sources, health agents can mobilize rapid response, reducing morbidity and mortality.	Disease surveillance based on online sources must be used with caution. Automatic identification of disease events using data from SM has to cope with inherent biases, ie, false-positive events, introduced through geographic or cultural variability in language and reporting when compared with reliable traditional surveillance methods.
22	Dalili Shoaei and Dastani 2020 (36)	The Role of Twitter During the COVID-19 Crisis: A Systematic Literature Review	17	Web of Science and PubMed: 2019–2020	24	COVID-19	Twitter	The data available on Twitter can be used as a source for identifying geographical areas at risk and the outbreak of COVID-19.	Emotional use of Twitter causes rapid spread of false opinions and information among individuals in the context of the digital world. Risk of low-quality health-related information.
23	Agrawal and Gupta 2020 (21)	The Utility of Social Media during an Emerging Infectious Diseases Crisis: A Systematic Review of Literature	6	PubMed, Google Scholar, and Cochrane Library: from 2012 to 2019	49	Ebola virus, Zika virus, Nipah virus, West Nile, Bird flu, H1N1 Influenza	Facebook, Twitter, YouTube, Instagram	The present systematic review supports the use of SM as an important medium for the clinicians, public health practitioners, and laypeople seeking health information for the detection of EIDs. SM allows the health agencies to guide the public during surveillance of an EID. Twitter may allow the detection of disease outbreaks through analysis of data generated by SM. Twitter data is able to detect moderately small outbreaks within a few days and for large outbreaks within hours.	The generalizability and reliability for all searches are unpredictable as people may not be searching the top posts alone, and misleading information may also be present along with the reliable information, which can misguide the users. Since SM do not have the capacity to replace traditional systems of gathering information about a disease, they should primarily support the existing traditional methods and be viewed as an extension of the traditional system rather than an alternative.
24	Alvarez-Galvez et al. 2021 (41)	Determinants of Infodemics During Disease Outbreaks: A Systematic Review	23	PubMed, Scopus, Medline, Embase, CINAHL, Sociological abstracts, Cochrane Library, and Web of Science: from 2002 to 2019	42	H1N1 Influenza, Ebola virus, Zika virus, Influenza, SARS-CoV, H7N9, Dengue fever	Twitter, Facebook, Instagram, WhatsApp, YouTube	Among determinants of infodemics communication channels (such as SM) were identified.	SM can be source of mis/disinformation.
25	Chen and Wang 2021 (42)	Social Media Use for Health Purposes: Systematic Review	269	12 databases through ProQuest and EBSCO, including MEDLINE, Academic Search Complete, PsycINFO, CINAHL, Psychology and Behavioral Science Collection, and Coronavirus Research Database.: from 2006 to 2020.	544	Unrestricted (particularly cited Zika virus and COVID-19)	Unrestricted. Cited Twitter, Facebook, WeChat, online forums, Sina Weibo, Reddit, YouTube, WhatsApp, Instagram and other platforms such as Pinterest, Yelp, and Yahoo! Answer.	SM data can provide an accurate prediction of disease outbreak case count. In addition to outbreak prediction, demographic and geographic data obtained from SM can inform medical research and practice of the characteristics of people who are at risk of being infected. Twitter is the most used SM platform for individual illness and disease outbreak surveillance.	Privacy concerns were raised related to using SM for health purposes. Gaps still exist in research and practice such as lacking an official guideline about privacy issues related to using SM in health research recruitment, lacking an approach to guarantee online informed consent, and researchers and potential participants lacking the awareness of the privacy risks of SM research recruitment.
26	Tsao et al. 2021 (53)	What social media told us in the time of COVID-19: a scoping review	577	PubMed, Scopus, PsycINFO: from 2019 to 2020	81	COVID-19	Google Trends, Baidu Search Index, Sina Weibo, Wikipedia, Twitter, YouTube, WhatsApp, Instagram, Facebook, Reddit, Wechat, TikTok	For COVID-19, SM can have a crucial role in disseminating health information and tackling infodemics and misinformation.	–
27	Gunasekeran et al. 2022 (29)	The Impact and Applications of Social Media Platforms for Public Health Responses Before and During the COVID-19 Pandemic: Systematic Literature Review	28	PubMed, Medline and Institute of Electrical and Electronics Engineers Xplore, from December 10, 2015, to December 10, 2020	678	Zika virus, H1N1 Influenza, Ebola virus, COVID-19, H7N9	TikTok, Facebook, Instagram, Twitter, Baidoo, Sina Weibo	The review emphasizes the importance of using SM platforms as useful applications for public health communication, monitoring, and predictions.	To be most effective, it is needed to develop a participatory approach involving members of target populations.
28	Khan et al. 2022 (33)	Artificial Intelligence and Internet of Things (AI-IoT) Technologies in Response to COVID-19 Pandemic: A Systematic Review	15	Not specified	325	COVID-19	Facebook, WhatsApp, Instagram, Twitter	SM-based predictions of epidemic statistics are needed to compensate for deficiencies of traditional epidemic data collection processes.	The massive amounts of information produced and exchanged on SM and websites, with its intensity overshot in the recent COVID-19 pandemic, increases the risk of significance of the associated demerit, i.e., false information. In this context, it is necessary to find solutions to detect misinformation online, reducing the risk of deviation of political, social and health education correctness of a large portion of the society, which can indirectly affect the efforts against containing the pandemic.
29	Takats et al. 2022 (45)	Ethical and Methodological Considerations of Twitter Data for Public Health Research: Systematic Review	10	SocINDEX, PsycINFO, and PubMed (articles published between January 2006 and October 31, 2019)	367	Influenza, Ebola virus, Zika virus	Twitter	Twitter data may be useful in public health research, given its access to publicly available information.	Health authorities who intend to use content from SM and other Internet data for public health surveillance need to consider protection and privacy, such as legal and ethical implications.
30	Campo et al. 2023 (54)	Use of twitter for health communication: a systematic review	0	Web of science, PubMed	83	COVID-19, Ebola virus, HIV, Influenza, Zika virus	Twitter	Twitter data may be useful for conversation and engagement during a pandemic.	There is no consensus on the usefulness of Twitter as a tool for information or for generating debate, although the platform’s effectiveness for measuring the impact of health campaigns was highlighted.
31	Pujante-Otalora et al. 2023 (55)	The use of networks in spatial and temporal computational models for outbreak spread in epidemiology: A systematic review	2	ACM Digital Library, IEEE Xplore, PubMed and Scopus databases, published between 2010 and September 2021	112	COVID-19, H1N1 Influenza, Ebola virus, MERS, Tubercolosis, MRSA, HFMD, SARS-CoV, Influenza	Unresticted	Social networks can be used as data source to collect information regarding the relationships among people.	–
32	Pilipiec et al. 2023 (56)	Surveillance of communicable diseases using social media: A systematic review	2	ACM Digital Library, IEEE Xplore, PubMed, and Web of Science: from 2010 to 2020	23	COVID-19, Dengue fever, Ebola virus, HIV, Influenza, Tubercolosis, Listeria, Measles	Sina Weibo, Twitter, Yahoo	SM can serve as a novel and powerful tool for the automated, real-time, and remote monitoring of public health and for the surveillance and prediction of communicable diseases in particular. Practitioners are highly recommended to include textual content from SM as a supplementary source for their data in their public health surveillance efforts to monitor and predict communicable diseases.	–

### 2.4 Quality assessment and risk of bias across studies

The quality of the included systematic reviews was assessed using the “A Measurement Tool to Assess systematic Reviews 2” (AMSTAR2) (20). AMSTAR2 comprises 16 elements that address the reviews’ design (i.e., *a priori*), data extraction, details of the literature search, inclusion of gray literature, characteristics, methods, the scientific quality of included studies, publication bias, and acknowledgement of conflict of interest(s). Each area in AMSTAR2 is investigated using “yes,” “partial yes,” or “no.” The quality assessment was conducted by two independent reviewers with a high agreement and then checked by a third one. To assign a final score to the selected studies, each systematic review received, respectively, a final rating of 1 for “yes,” 0,5 for “partial yes” and 0 for “no.” We assigned a score equal to 0 whether an item was not reported, not specified or unclear. For “yes” and “partial yes” the AMSTAR2 checklist provides specific elements (sub-items) which a systematic review should include. For items 2 and 4, specific sub-items were highlighted, and the score was calculated by dividing the main item score by the total number of sub-items. The only change introduced in the AMSTAR2 checklist concerns the item 16: a “Partial Yes” section was introduced to account for studies that reported either conflicts of interest, funding sources, or both. Then, AMSTAR2 results were summarized into an overall score out of 16.

The quality assessment revealed that most reviews met the essential AMSTAR2 criteria, including clear research questions, comprehensive search strategies, and well-defined inclusion and exclusion criteria.

However, some concerns were raised about the lack of standardized risk-of-bias assessments in certain reviews, which could affect the reliability of the synthesized evidence. The overall quality of the included reviews ranged from moderate to high, with only a few scoring lower in terms of methodological rigor.

Detailed results of the AMSTAR2 assessment for each review are provided in Supplementary material 2 (pages 13–21).

## 3 Results

### 3.1 Flow diagram

**Figure 1:**
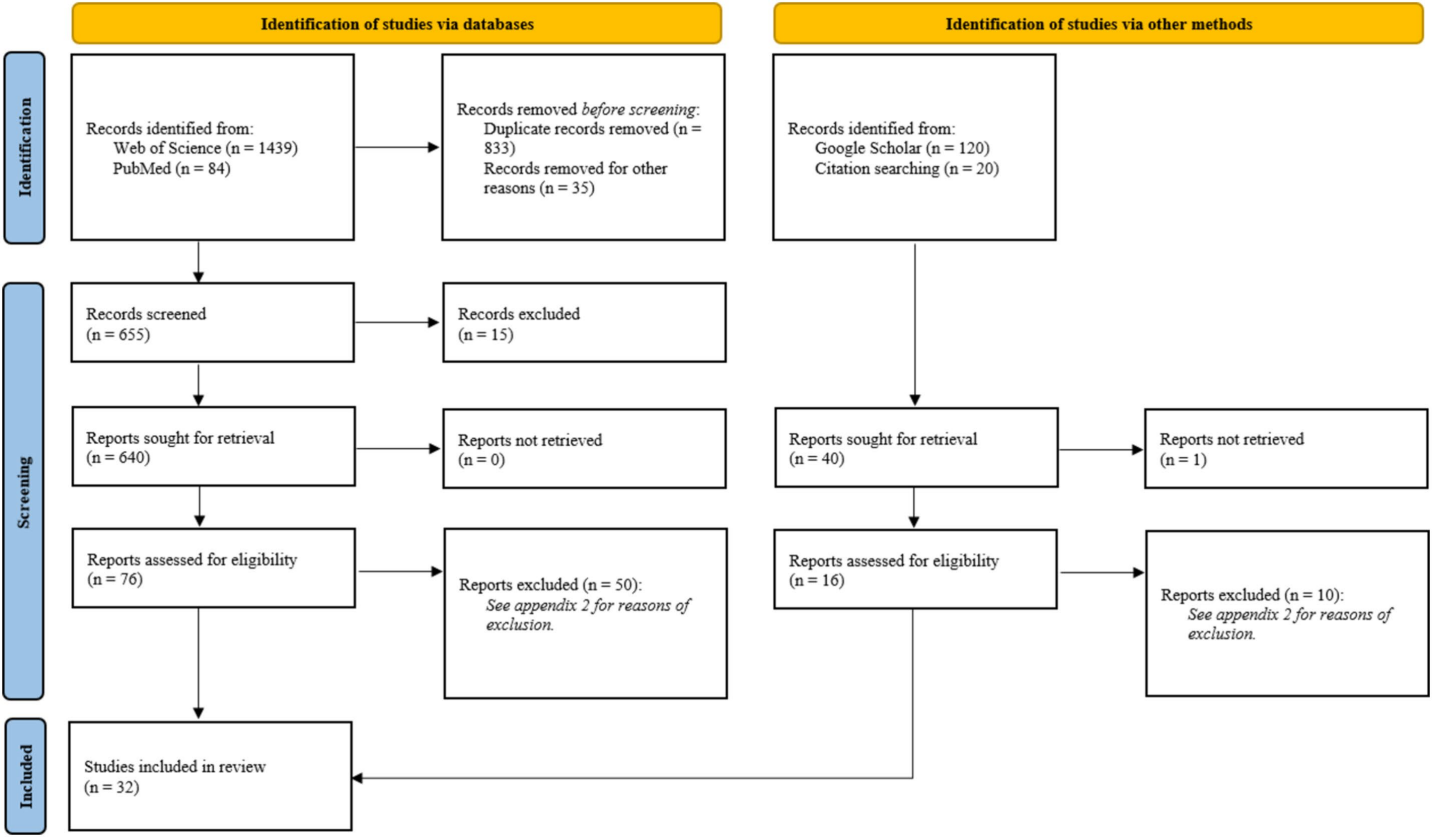
Prisma 2020 flow diagram from Page et al. (52). For more information, visit: http://www.prisma-statement.org/.

The initial search yielded 1,663 articles (Figure 1). After reviewing titles and abstracts, 86 studies were selected, which were further narrowed down to 32 review articles after full-text evaluation. The excluded studies and the reasons for exclusion are listed in Supplementary Table 2.

### 3.2 Temporal extension and journals

**Figure 2:**
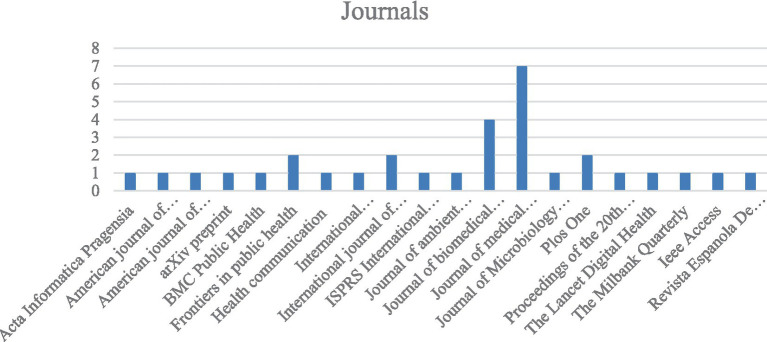
Journal list. Source: Authors’ elaboration.

As digital epidemiology emerged in the 21st century, no selected articles in this study were published before 2011. From 2011 to 2015, 7 systematic reviews were included. In 2016, three papers were found, followed by three reviews each in 2017 and 2018. One paper was published in 2019, five in 2020, and four in 2021. Finally, three reviews were published in both 2022 and 2023. Figure 2 presents a vertical histogram showing the journals with the highest publication frequency.

### 3.3 Key findings in using social media for disease surveillance

Starting from the systematic reviews, this study aimed to explore how SM can improve the early detection of infectious disease outbreaks.

The review studies’ results (see Table 2) can be summarized as follows:

- SM is effective in supporting public health efforts in identifying EIDs and target populations for interventions. Agrawal and Gupta (21), for example, confirm the potentiality of the SM data for the clinicians, public health practitioners, and lay people that seek health information for the detection of EIDs. Also Gianfredi et al. (22), consider data streams as promising tools for predicting the spread of infectious agents, especially when information and parameters related to the infection rates are scarcely known or not available.
- This emphasizes the advantages of integrating SM analyses into traditional disease surveillance and outbreak management practices (23–29): although social networks will probably never replace traditional surveillance systems, the integration can offer complementary data, improving the disease prediction ability of traditional syndromic surveillance systems (30–33). However, integration with traditional data requires advanced algorithms and computational linguistics methods (3).
- SM can be used for geolocalizing potential chains of contagion. Luan and Law (34) showed that SM can enhance investigations for timely collection of geo-referenced health data, aiding large-scale public health surveillance. Carroll et al. (35) concluded that social networks could be useful in identifying the source case and predicting which individuals are more likely to become infected and further infect others (35). Also Dalili Shoaei and Dastani (36), confirmed that the information drawn from SM platforms can provide useful demographic and geographic details on the characteristics of people who are at potential risk of being infected (36). In this context, Twitter platform is the most used social network (45, 49).
- SM listening can be used as a communication tool available for public policy makers, especially in the initial stages of the infection spread. Eckert et al. (37), for example, demonstrated the successful use of SM by government agencies to share accurate information and debunk misinformation, particularly during the preparation, onset, and containment phases. However, Fung et al. (38) noted that it may occasionally fail, as seen during the Ebola virus disease emergency response.
- There are some limitations in using a social network as a surveillance instrument. These are related to:

- misinformation phenomena (33, 39–41);
- lack of an ethical framework (25, 30, 32);
- lack of internationally recognized policies regarding privacy protection (30–32, 42–45);
- risk of dissemination of conspiracy theories, pseudo-scientific claims and misleading information, particularly during the initial phase of the pandemic (22, 36);
- extraction - given the heterogeneous data characteristics - of unstructured data from SM (27, 30, 46, 47);
- biases in terms of minor accuracy, i.e., false-positive events introduced through geographic or cultural variability in language and reporting when compared with reliable traditional surveillance methods (28).

### 3.4 The role of the social media in detecting each disease: the circle packing visualization and a network analysis

**Figure 3:**
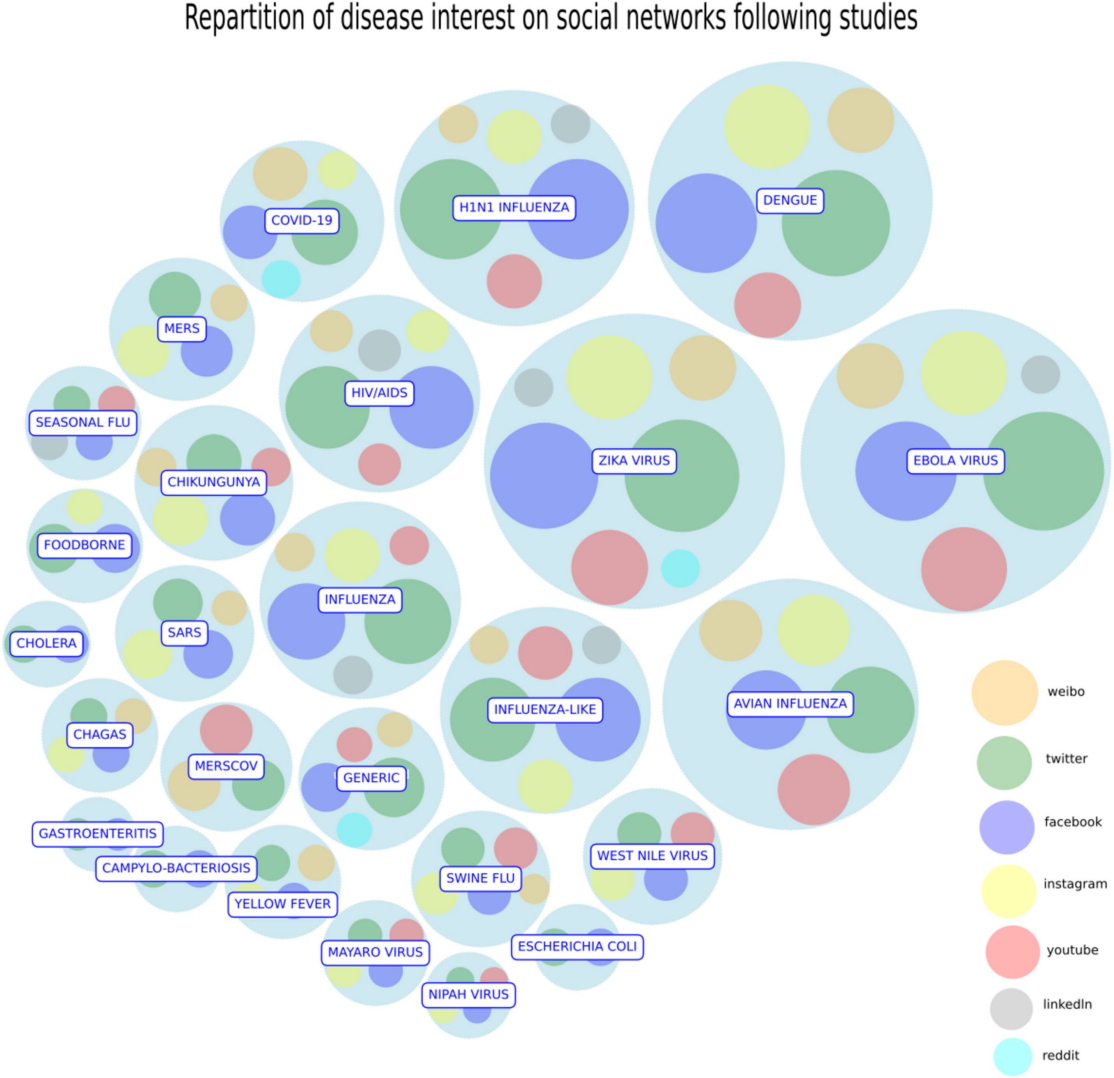
Circlepack plot. The *circle pack plot* represents the importance of each disease and the SM citing it as nested circles. Containers circles describe each disease proportional to the number of citations in studies, while for each disease the relative importance of each SM is expressed by the radius of their inner circle. Date reference: October 2023. Source: Authors’ elaboration.

In this paragraph, we investigate the studies selected in our systematic review, starting from the following research question: *Is it possible to explore the role and thus the importance of a specific SM to deal with a disease?* To visualize the importance of each disease and SM tools in tracking and monitoring the disease, we use a circlepack plot (Figure 3): a tool that uses nested circles proportional to the number of citations of, respectively, each disease (container circles) and each SM (inner circles) citing the disease.

**Figure 4:**
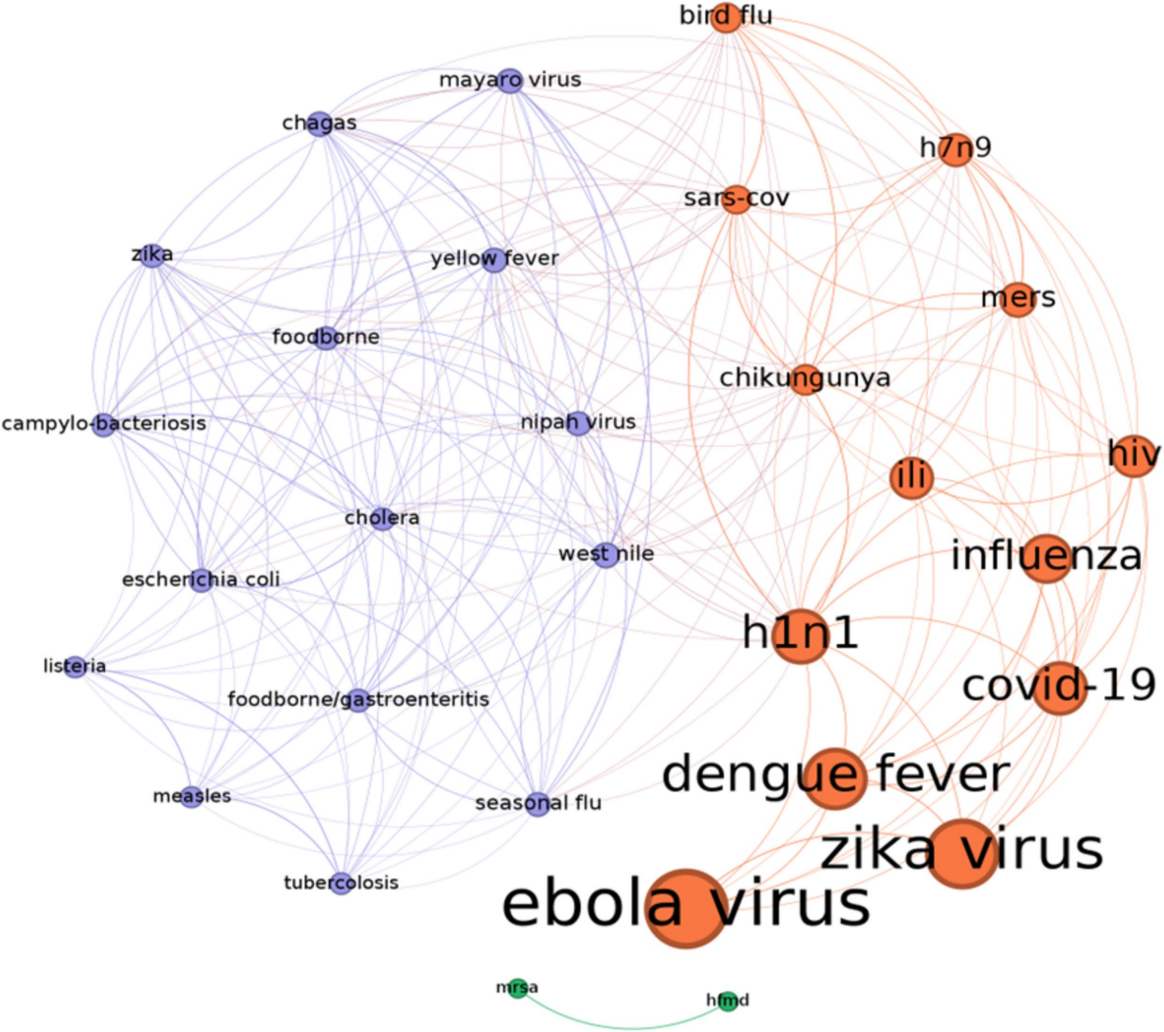
The diseases’ similarity network according to their citations on social networks. In detail, the more two diseases share a similar impact on different social networks the stronger is their link (shown by thickness of the link). The size of nodes is proportional to the number of citations. The color of the nodes defines three communities dividing the diseases in two main groups that received more homogeneous coverage by social networks, and a disconnected sub-network connecting mrsa, and hfmd (hfmd: hand foot and mouth diseases, mrsa: methicillin-resistant *staphylococcus aureus*). Source: Authors’ elaboration.

In this way, we can both summarize how the literature studies and weights each SM in the analysis of every disease. After conducting the analysis, it was found that the number of studies discussing the COVID-19 pandemic is consistent and equally important as other relevant diseases. However, they have not yet reached the highest position in the ranking list because of a possible “delay effect.” In a future, it is expected that a significant number of papers on COVID-19 will be published. On the other side, Zika, Ebola and the influenza H1N1, Dengue Fever, together with COVID-19 show the largest bubble sizes since they have raised a lot of concern in the public and they were vastly studied by the experts of the field as the first examples of disease communication through the social networks. Twitter, Facebook, Youtube and Instagram are the most relevant social platforms in the circlepack plot. Other social platforms of less importance, like MySpace, show a smaller size bubble: although they pioneered the initial phase of the internet diffusion, they are now extinct. Finally, Weibo and VK have a minor circle size because they target specific markets (Chinese for Weibo and Russian for VK). To complete the analysis, we propose a network representation of the diseases according to the intensity of the social networks’ communication (Figure 4). The basic idea is to represent diseases according to the way SM treat them. Two diseases form a link if they are similar in the treatment they received by SM. This measure represents both citation impact (size of the node) and visual representation of SM response.

The steps of the network formation are the following:

- Each disease is linked to a vector of social networks with vector elements that correspond to a number of studies’ citations per disease;
- The links reflect how many social networks, and corresponding citations, two diseases have in common. If two diseases share a similar presence on social networks, the link becomes stronger. The used similarity metric is the “weighted Jaccard index”.^1^
- Links are removed below a given threshold (0.3) to maintain only the stronger levels of similarity;
- Nodes were colored using a community detection algorithm (48) that recovers the homogeneous disease by dividing nodes into dense groups.

The number of studies on each disease determines its relevance in the network structure. Ebola received the most attention, followed by Zika, Dengue, and COVID-19.

A simple clustering procedure, known as community detection (48) is then used to divide the nodes in groups/clusters that are homogenous according to how the social networks discuss a particular disease (Figure 3).

According to this representation, the cluster of “flu like” diseases and viruses is identified in orange color, opposed to a cluster of different diseases with cholera, tuberculosis and other gastroenteritis viruses representing a different sample set that is shown in violet. In summary, we recover the importance of COVID-19 through the higher number of citations making it among the most discussed diseases (it is not surprising) in the social networks’ studies. Ebola virus, identified in 1976, remains a deadly disease with high mortality rates capturing a growing attention of the researchers and the discussions on virtually all social networks.

## 4 Discussion

This umbrella review included 32 systematic reviews and 3,704 primary studies on the use of SM to detect EIDs. While policymakers should be cautious about the limitations of using digital data for health surveillance, most studies highlight positive outcomes. SM plays a key role in policy actions and healthcare management, helping reduce morbidity and mortality. It provides an opportunity to detect early epidemic signals, complementing traditional surveillance systems, which, though specific, often face delays, high costs, and geographical limitations.

Our review has both strengths and limitations.

A major strength is the comprehensive overview of SM’s role in detecting emerging diseases, covering a wide range of diseases and social networks. The systematic approach highlights new insights into SM’s role in health surveillance. For instance, only a few studies, such as Velasco et al. (32), focus on event-based approaches, whereas most rely on continuous SM news streams (filtered by keywords, like disease symptoms such as “cough,” “fever” etc.), which can be misleading and imprecise. Targeting specific events (e.g., outbreak news) could improve robustness and reduce misinformation. Additionally, De Araujo et al. (27) suggest using SM as active tools, connecting experts and practitioners to create geographical networks for verifying health emergencies.

Some limitations should be noted. First, the scope of the review was limited to systematic reviews published up to October 2023, which may have excluded relevant studies published thereafter. This temporal limitation might affect the comprehensiveness of the findings, particularly given the rapidly evolving nature of studies on this topic. Second, non-English papers were excluded, potentially missing relevant studies and excluding evidence from countries with different policy approaches. Third, we did not assess the quality of the primary studies, which was the role of the original systematic reviews. Also, although SM shows potential as a complement to traditional systems, challenges such as misinformation, noise, and privacy concerns remain. The unstructured nature of SM data complicates its integration into existing surveillance systems, requiring further research to address these issues. Finally, we attempted to minimize sample bias through a systematic search for gray literature using Google Scholar; however, we cannot fully exclude it due to our limited search.

It is also important to acknowledge the limitations inherent in many of the systematic reviews included. Many studies used observational or retrospective data, leading to potential biases in data collection and interpretation. Most primary studies relied on self-reported or public SM data, which may be unrepresentative and subject to selection bias. Moreover, methodological heterogeneity made it difficult to compare findings across reviews, with different computational tools, algorithms, and linguistic methods producing inconsistent outcomes. This variability also applied to the types of SM platforms studied, with more popular platforms like Twitter and Facebook receiving more attention, while others potentially relevant to specific populations were overlooked.

## 5 Conclusion and implications

We can conclude that the policy interventions strongly benefit from the continued use of online data in public health surveillance systems, in order to improve the disease prediction abilities of the traditional surveillance systems.

The findings emphasize several key policy implications. First, SM analytics are transforming how public health experts monitor diseases by integrating real-time geolocation of health status, allowing early identification of potential pandemics.

Second, for health systems management, the digital innovation based on SM is a tool to promote new models of prevention, diagnosis, treatment and assistance that are sustainable and interconnected with the transformation processes underway (reduction of resources, demographic explosion, aging of the population, affirmation of new health emergencies connected to the phenomenon of globalization).

These transformations are the basis of that new frontier of eHealth which refers to the combined use of electronic communication and technological information analytics in the health sector (49). Finally, the detection of early outbreaks through SM tracking provides a significant timeliness advantage in a variety of EIDs, potentially improving healthcare systems efficiency in terms of a better management of healthcare facilities.

Policy makers and governments may effectively leverage SM information if they implement research projects and specialized platforms. These ones should perform social listening of disease symptoms for early outbreak detection, and starting from social interaction data, help creating specialized networks of professionals (doctors, scientists and others) that will cope with misinformation and will provide firsthand evidence of emerging outbreaks.

Overall, misinformation for the vast public can be counteracted by working together with SM platforms to promote curated content, reducing user exposure to fake news (50, 51). In this context, policymakers can support initiatives targeted to measure the effects of correct information, like for example the user polarization caused by specific fake news on a given topic, does it really decrease after an information campaign?

This study aims to provide helpful suggestions to health policy decision-makers in deciding whether to incorporate new methods into comprehensive programs of surveillance that already contain well established indicator-based surveillance tools. Considering the limitations associated with the use of SM data, we suggest the creation of specially designated working groups able to analyze and correctly make use of the online data, to reduce the potential of SM into spreading rumors, fake news, and misleading information. From this point of view, cost–benefit investigations are needed to examine the advantages of SM data in the long term, guiding future best practices in the field. The creation of an integrated, accessible, and constantly updated data set is the first step toward the creation of both an effective and efficient pandemic surveillance system. It will not only be fed by data from digital platforms, but multiple sources can also be relevant to build a multisectoral database. As possible inputs, it may also include environmental data (i.e., composition of wastewater that may contain traces of the disease pathogens), which, whether properly analyzed, can provide useful information to prevent the emergence of new pathogens.

## Data Availability

The original contributions presented in the study are included in the article/Supplementary material, further inquiries can be directed to the corresponding author.

## Footnotes

^1^ https://rpubs.com/lgadar/weighted-jaccard
