# Supplementary material for: Innovating health prevention models in detecting infectious disease outbreaks through social media data: an umbrella review of the evidence
Source: Front Public Health. 2024 Nov 22;12:1435724. doi: 10.3389/fpubh.2024.1435724 (PMC11621043; doi:10.3389/fpubh.2024.1435724)
Supplement: Supplementary file 1 [file Table_1.docx]

**SUPPLEMENTARY MATERIAL 1.** *TABLE 1:* Prisma 2020 Checklist & PRISMA Abstract checklist. *Table 2:* Excluded studies

**Table 1.** PRISMA 2020 Checklist

| **Section and Topic** | **Item #** | **Checklist item** | **Location where item is reported** |
| --- | --- | --- | --- |
| **TITLE** | | |  |
| Title | 1 | Identify the report as a systematic review. | Title - Pag. 1 (Umbrella review) |
| **ABSTRACT** | | |  |
| Abstract | 2 | Provide a structured summary including, as applicable: background; objectives; data sources; study eligibility criteria, participants, and interventions; study appraisal and synthesis methods; results; limitations; conclusions and implications of key findings; systematic review registration number. | Pag.1 |
| **INTRODUCTION** | | |  |
| Rationale | 3 | Describe the rationale for the review in the context of existing knowledge. | Pag. 2 |
| Objectives | 4 | Provide an explicit statement of the objective(s) or question(s) the review addresses. | Pag. 22 |
| **METHODS** | | |  |
| Eligibility criteria | 5 | Specify the inclusion and exclusion criteria for the review and how studies were grouped for the syntheses. | Pag. 3 |
| Information sources | 6 | Specify all databases, registers, websites, organisations, reference lists and other sources searched or consulted to identify studies. Specify the date when each source was last searched or consulted. | Pag. 3 |
| Search strategy | 7 | Present the full search strategies for all databases, registers and websites, including any filters and limits used. | Pag. 3 |
| Selection process | 8 | Specify the methods used to decide whether a study met the inclusion criteria of the review, including how many reviewers screened each record and each report retrieved, whether they worked independently, and if applicable, details of automation tools used in the process. | Pag. 3 |
| Data collection process | 9 | Specify the methods used to collect data from reports, including how many reviewers collected data from each report, whether they worked independently, any processes for obtaining or confirming data from study investigators, and if applicable, details of automation tools used in the process. | Pag. 3 |
| Data items | 10a | List and define all outcomes for which data were sought. Specify whether all results that were compatible with each outcome domain in each study were sought (e.g. for all measures, time points, analyses), and if not, the methods used to decide which results to collect. | Pag. 3 |
|  | 10b | List and define all other variables for which data were sought (e.g. participant and intervention characteristics, funding sources). Describe any assumptions made about any missing or unclear information. | Pag. 3 |
| Study risk of bias assessment | 11 | Specify the methods used to assess risk of bias in the included studies, including details of the tool(s) used, how many reviewers assessed each study and whether they worked independently, and if applicable, details of automation tools used in the process. | Pag. 3 |
| Effect measures | 12 | Specify for each outcome the effect measure(s) (e.g. risk ratio, mean difference) used in the synthesis or presentation of results. | Pag. 3 |
| Synthesis methods | 13a | Describe the processes used to decide which studies were eligible for each synthesis (e.g. tabulating the study intervention characteristics and comparing against the planned groups for each synthesis (item #5)). | Pag. 3 |
|  | 13b | Describe any methods required to prepare the data for presentation or synthesis, such as handling of missing summary statistics, or data conversions. | Pag. 3 |
|  | 13c | Describe any methods used to tabulate or visually display results of individual studies and syntheses. | Pag. 3 |
|  | 13d | Describe any methods used to synthesize results and provide a rationale for the choice(s). If meta-analysis was performed, describe the model(s), method(s) to identify the presence and extent of statistical heterogeneity, and software package(s) used. | Pag. 3 |
|  | 13e | Describe any methods used to explore possible causes of heterogeneity among study results (e.g. subgroup analysis, meta-regression). | Pag. 3 |
|  | 13f | Describe any sensitivity analyses conducted to assess robustness of the synthesized results. | Pag. 3 |
| Reporting bias assessment | 14 | Describe any methods used to assess risk of bias due to missing results in a synthesis (arising from reporting biases). | Pag. 3 |
| Certainty assessment | 15 | Describe any methods used to assess certainty (or confidence) in the body of evidence for an outcome. | Pag. 3 |
| **RESULTS** | | |  |
| Study selection | 16a | Describe the results of the search and selection process, from the number of records identified in the search to the number of studies included in the review, ideally using a flow diagram. | Pag. 14 – Figure 1 |
|  | 16b | Cite studies that might appear to meet the inclusion criteria, but which were excluded, and explain why they were excluded. | Pag. 14; Supplementary Material 1 – Table 2 |
| Study characteristics | 17 | Cite each included study and present its characteristics. | Pag. 4-13, Table 2 |
| Risk of bias in studies | 18 | Present assessments of risk of bias for each included study. | Supplementary Material 2 (AMSTAR 2 – Quality assessment) |
| Results of individual studies | 19 | For all outcomes, present, for each study: (a) summary statistics for each group (where appropriate) and (b) an effect estimate and its precision (e.g. confidence/credible interval), ideally using structured tables or plots. | Pag. 14-17 |
| Results of syntheses | 20a | For each synthesis, briefly summarise the characteristics and risk of bias among contributing studies. | Pag. 14-17 |
|  | 20b | Present results of all statistical syntheses conducted. If meta-analysis was done, present for each the summary estimate and its precision (e.g. confidence/credible interval) and measures of statistical heterogeneity. If comparing groups, describe the direction of the effect. | Pag. 14-17 |
|  | 20c | Present results of all investigations of possible causes of heterogeneity among study results. | Pag. 14-17 |
|  | 20d | Present results of all sensitivity analyses conducted to assess the robustness of the synthesized results. | NA |
| Reporting biases | 21 | Present assessments of risk of bias due to missing results (arising from reporting biases) for each synthesis assessed. | NA |
| Certainty of evidence | 22 | Present assessments of certainty (or confidence) in the body of evidence for each outcome assessed. | NA |
| **DISCUSSION** | | |  |
| Discussion | 23a | Provide a general interpretation of the results in the context of other evidence. | Pag. 17-18 |
|  | 23b | Discuss any limitations of the evidence included in the review. | Pag. 17-18 |
|  | 23c | Discuss any limitations of the review processes used. | Pag. 17-18 |
|  | 23d | Discuss implications of the results for practice, policy, and future research. | Pag. 17-18 |
| **OTHER INFORMATION** | | |  |
| Registration and protocol | 24a | Provide registration information for the review, including register name and registration number, or state that the review was not registered. | (PROSPERO ID: CRD42021254568) - Pag. 1 |
|  | 24b | Indicate where the review protocol can be accessed, or state that a protocol was not prepared. | Pag. 1, 2 |
|  | 24c | Describe and explain any amendments to information provided at registration or in the protocol. | Pag. 2 |
| Support | 25 | Describe sources of financial or non-financial support for the review, and the role of the funders or sponsors in the review. | Pag. 18 |
| Competing interests | 26 | Declare any competing interests of review authors. | NA |
| Availability of data, code and other materials | 27 | Report which of the following are publicly available and where they can be found: template data collection forms; data extracted from included studies; data used for all analyses; analytic code; any other materials used in the review. | Pag. 1, 2– PROSPERO database |

*From:*  Page MJ, McKenzie JE, Bossuyt PM, Boutron I, Hoffmann TC, Mulrow CD, et al. The PRISMA 2020 statement: an updated guideline for reporting systematic reviews. BMJ 2021;372:n71. doi: 10.1136/bmj.n71. For more information, visit: <http://www.prisma-statement.org/> . **NA:** Not applicable

**PRISMA 2020 – Abstract checklist**

| **Section and Topic** | **Item #** | **Checklist item** | **Reported (Yes/No)** |
| --- | --- | --- | --- |
| **TITLE** | | |  |
| Title | 1 | Identify the report as a systematic review. | Yes |
| **BACKGROUND** | | |  |
| Objectives | 2 | Provide an explicit statement of the main objective(s) or question(s) the review addresses. | Yes |
| **METHODS** | | |  |
| Eligibility criteria | 3 | Specify the inclusion and exclusion criteria for the review. | Yes |
| Information sources | 4 | Specify the information sources (e.g. databases, registers) used to identify studies and the date when each was last searched. | Yes |
| Risk of bias | 5 | Specify the methods used to assess risk of bias in the included studies. | Yes |
| Synthesis of results | 6 | Specify the methods used to present and synthesise results. | Yes |
| **RESULTS** | | |  |
| Included studies | 7 | Give the total number of included studies and participants and summarise relevant characteristics of studies. | Yes |
| Synthesis of results | 8 | Present results for main outcomes, preferably indicating the number of included studies and participants for each. If meta-analysis was done, report the summary estimate and confidence/credible interval. If comparing groups, indicate the direction of the effect (i.e. which group is favoured). | Yes |
| **DISCUSSION** | | |  |
| Limitations of evidence | 9 | Provide a brief summary of the limitations of the evidence included in the review (e.g. study risk of bias, inconsistency and imprecision). | Yes |
| Interpretation | 10 | Provide a general interpretation of the results and important implications. | Yes |
| **OTHER** | | |  |
| Funding | 11 | Specify the primary source of funding for the review. | NA |
| Registration | 12 | Provide the register name and registration number. | Yes |

**Table 2. Excluded studies**

| **N** | **Citation** | **Reason of exclusion** |
| --- | --- | --- |
| 1 | Ab Ghani, N. L., Mohd Drus, S., Hassan, N. H., & Abdul Latif, A. (2017). Factors of emerging infectious disease outbreak prediction using big data analytics: A systematic literature review. Proceedings of the 6 th International Conference on Computing and Informatics, ICOCI 2017 | Proceeding paper/Draft |
| 2 | Abkenar, S. B., Kashani, M. H., Mahdipour, E., & Jameii, S. M. (2020). Big data analytics meets social media: A systematic review of techniques, open issues, and future directions. Telematics and Informatics, 101517. | In this paper, authors demonstrate how big data analytics meets social media, and a comprehensive review is provided on big data analytic approaches in social networks to search published studies between 2013 and August 2020, with 74 identified papers. The study doesn't meet the aim of this systematic review. |
| 3 | Abkenar, S. B., Kashani, M. H., Mahdipour, E., & Jameii, S. M. (2020). Big data analytics meets social media: A systematic review of techniques, open issues, and future directions. Telematics and Informatics, 101517. | In this paper, authors demonstrate how big data analytics meets social media. The topic doesn't meet the scope of our systematic review. Only one study included was relevant considering our aims (Zadeh et al. 2019), but this study was comprised in the paper of Corsi et al. 2020. This last is part of selected systematic reviews in the present study. |
| 4 | Agheneza, Tumacha. A systematic literature review on even-based public health surveillance systems. PhD diss., Hochschule für angewandte Wissenschaften Hamburg, 2012. | Phd dissertation |
| 5 | Alamoodi, A., Zaidan, B., Zaidan, A., Albahri, O., Mohammed, K., Malik, R., ... & Alaa, M. (2020). Sentiment analysis and its applications in fighting COVID-19 and infectious diseases: A systematic review. Expert systems with applications, 114155. | The study examines the role of sentiment analysis in the occurrence of COVID-19 and other previous infectious diseases. It doesn't meet the scope of this systematic review. |
| 6 | Alshaikh, F., Ramzan, F., Rawaf, S., & Majeed, A. (2014). Social network sites as a mode to collect health data: a systematic review. Journal of medical Internet research, 16(7), e171. | The objective of this study was to systematically review the available literature and explore the use of social network sites as a mode of collecting data for health research. It has no connection to the use of social media in detecting emerging infectious diseases. Accordingly, it doesn't meet the scope of this systematic review. |
| 7 | Aydin, G. (2020). Improving health and efficiency with strategic social media use in health organizations: a critical review of the status quo. Multidimensional Perspectives and Global Analysis of Universal Health Coverage, 309-336. | Book chapter |
| 8 | Bajpai, A., & Sharma, S. (2018). Big data analysis in health care domain: a systematic review. International Journal of Engineering Technologies and Management Research, 5(2), 1-8. | This paper provides the review of various research disciplines made in health care realm using big data approaches and methodologies. It has no connection to social media and infectious disease outbreak. Accordingly, it doesn't meet the scope of this systematic review. |
| 9 | Bou-Karroum, L., El-Jardali, F., Hemadi, N., Faraj, Y., Ojha, U., Shahrour, M., ... & Akl, E. A. (2017). Using media to impact health policy-making: an integrative systematic review. Implementation Science, 12(1), 1-14. | This is a systematic review aimed to assess the effects of planned media interventions - including social media - on the health policy-making process. It has no connection to social media and infectious disease outbreak. Accordingly, it doesn't meet the scope of this systematic review. |
| 10 | Bravata, D. M., McDonald, K. M., Smith, W. M., Rydzak, C., Szeto, H., Buckeridge, D. L., ... & Owens, D. K. (2004). Systematic review: surveillance systems for early detection of bioterrorism-related diseases. Annals of internal medicine, 140(11), 910-922. | This systematic review critically evaluates the potential utility of existing surveillance systems for illnesses and syndromes related to bioterrorism. It has no connection to social media and it doesn't meet the scope of our study. |
| 11 | Brinkel, J., Krämer, A., Krumkamp, R., May, J., & Fobil, J. (2014). Mobile phone-based mHealth approaches for public health surveillance in sub-Saharan Africa: a systematic review. International journal of environmental research and public health, 11(11), 11559-11582. | The objective of this review was to examine mobile phone-based mHealth interventions for Public Health surveillance in sub-Sahara Africa. It has no connection to social media and it doesn't meet the scope of our study. |
| 12 | Calba, C., Goutard, F. L., Hoinville, L., Hendrikx, P., Lindberg, A., Saegerman, C., & Peyre, M. (2015). Surveillance systems evaluation: a systematic review of the existing approaches. BMC public health, 15(1), 1-13. | The purposes of this review were therefore to identify and analyse the existing health surveillance systems evaluation approaches in order to allow end users (i.e. decision makers in health surveillance programs at all administrative levels of implementation) to select the most appropriate approach based on their objective(s) and also to inform the development of an evaluation framework within the RISKSUR project a (Risk-based animal health surveillance systems). It has no connection to social media and infectious disease outbreak. Accordingly, it doesn't meet the scope of this systematic review. |
| 13 | Cao, B., Gupta, S., Wang, J., Hightow-Weidman, L. B., Muessig, K. E., Tang, W., ... & Tucker, J. D. (2017). Social media interventions to promote HIV testing, linkage, adherence, and retention: systematic review and meta-analysis. Journal of medical Internet research, 19(11), e394. | This systematic review examines the effectiveness of social media interventions to promote HIV testing, linkage, adherence, and retention among key populations. It doesn't meet the scope of this systematic review. |
| 14 | Capurro, D., Cole, K., Echavarría, M. I., Joe, J., Neogi, T., & Turner, A. M. (2014). The use of social networking sites for public health practice and research: a systematic review. Journal of medical Internet research, 16(3), e79. | Authors conducted a systematic review to identify the use of social networking sites for public health research and practice and to identify existing knowledge gaps. Articles selected describe different approaches to social networking sites. It has no direct connection to social media and infectious disease outbreak. Only two studies included were relevant considering our aims (Chew et al., 2020, and Signorini et al., 2011), but these studies were comprised in the paper of Charles-Smith et al., 2015. This last is part of selected systematic reviews in the present study. |
| 15 | Cavus, N., Sani, A. S., Haruna, Y., & Lawan, A. A. (2021). Efficacy of Social Networking Sites for Sustainable Education in the Era of COVID-19: A systematic review. Sustainability, 13(2), 808. | The current study systematically reviewed recent articles that recognized the value and feasibility of using Social Networking Sites (SNSs) in education. It doesn't meet the scope of this systematic review. |
| 16 | Chambers, D., Wilson, P., Thompson, C., & Harden, M. (2012). Social network analysis in healthcare settings: a systematic scoping review. PloS one, 7(8), e41911. | Authors conducted a systematic scoping review to identify and evaluate the use of social network analysis as part of an intervention to support the implementation of change in healthcare settings. It has no connection to the use of social media in detecting emerging infectious diseases. Accordingly, it doesn't meet the scope of this systematic review. |
| 17 | Cheston, C. C., Flickinger, T. E., & Chisolm, M. S. (2013). Social media use in medical education: a systematic review. Academic Medicine, 88(6), 893-901. | The authors conducted a systematic review of the published literature on social media use in medical education. It has no connection to the use of social media in detecting emerging infectious diseases. Accordingly, it doesn't meet the scope of this systematic review. |
| 18 | Clark, R. C., & Mytton, J. (2007). Estimating infectious disease in UK asylum seekers and refugees: a systematic review of prevalence studies. Journal of Public Health, 29(4), 420-428. | Authors conducted a systematic review of observational studies to identify research findings reporting the prevalence of tuberculosis, HIV and hepatitis B in the UK asylum seeker and refugee population. It has no connection to the use of social media in detecting emerging infectious diseases such as Zika virus, Ebola, etc. Accordingly, it doesn't meet the scope of this systematic review. |
| 19 | Davalbhakta, S., Advani, S., Kumar, S., Agarwal, V., Bhoyar, S., Fedirko, E., ... & Agarwal, V. (2020). A systematic review of smartphone applications available for corona virus disease 2019 (COVID19) and the assessment of their quality using the mobile application rating scale (MARS). Journal of medical systems, 44(9), 1-15. | Authors performed a systematic review of the literature and mobile platforms to assess mobile applications currently utilized for COVID-19, and a quality assessment of these applications using the Mobile Application Rating Scale (MARS) for overall quality, Engagement, Functionality, Aesthetics, and Information. It has no connection to the use of social media in detecting emerging infectious diseases. Accordingly, it doesn't meet the scope of this systematic review. |
| 20 | Dedeilia, A., Sotiropoulos, M. G., Hanrahan, J. G., Janga, D., Dedeilias, P., & Sideris, M. (2020). Medical and surgical education challenges and innovations in the COVID-19 era: a systematic review. in vivo, 34(3 suppl), 1603-1611. | The aim of this systematic review was to identify the challenges imposed on medical and surgical education by the COVID-19 pandemic. It includes the role of social media but medical education is not the focus of our study. |
| 21 | Déglise, C., Suggs, L. S., & Odermatt, P. (2012). SMS for disease control in developing countries: a systematic review of mobile health applications. Journal of Telemedicine and Telecare, 18(5), 273-281. | Authors examined SMS - supported interventions for prevention, surveillance, management and treatment compliance of communicable and non- communicable diseases in developing countries. It has no connection to the use of social media in detecting emerging infectious diseases. Accordingly, it doesn't meet the scope of this systematic review. |
| 22 | Ford, E., Shepherd, S., Jones, K., & Hassan, L. (2021). Toward an Ethical Framework for the Text Mining of Social Media for Health Research: A Systematic Review. Frontiers in Digital Health, 2, 62. | Authors conducted a systematic review on the use of social media text by users to acquire information about their health. It has no connection to the use of social media in detecting emerging infectious diseases. Accordingly, it doesn't meet the scope of this systematic review. |
| 23 | Franco, M., Tursunbayeva, A., & Pagliari, C. (2016). Social media for e-Government in the public health sector: protocol for a systematic review. JMIR research protocols, 5(1), e5421. | A systematic literature review is underway to capture and synthesize existing evidence on the adoption, use, and impacts of social media in the public health sector. The review doesn't not include insights about the use of social media in detecting emerging infectious diseases. Accordingly, it doesn't meet the scope of this systematic review. |
| 24 | Fung, I. C. H., Blankenship, E. B., Ahweyevu, J. O., Cooper, L. K., Duke, C. H., Carswell, S. L., ... & Tse, Z. T. H. (2020). Public health implications of image-based social media: A systematic review of Instagram, Pinterest, Tumblr, and Flickr. The Permanente Journal, 24. | Authors conducted a systematic review to better understand the use of social media as tool to explore health communication. The review doesn't not include insights about the use of social media in detecting emerging infectious diseases. Accordingly, it doesn't meet the scope of this systematic review. |
| 25 | Gaitanou, P., Garoufallou, E., & Balatsoukas, P. (2014, November). The effectiveness of big data in health care: a systematic review. In Research conference on metadata and semantics research (pp. 141-153). Springer, Cham. | The aim of the study was to perform a systematic review of the literature in order to determine the extent to which Big Data applications in health care systems have managed to improve patient experiences and clinicians' behavior as well as the quality of care provided to patients. It has no connection to the use of social media in detecting emerging infectious diseases. Accordingly, it doesn't meet the scope of this systematic review. |
| 26 | Gajewski, K. N., Peterson, A. E., Chitale, R. A., Pavlin, J. A., Russell, K. L., & Chretien, J. P. (2014). A review of evaluations of electronic event-based biosurveillance systems. PloS one, 9(10), e111222. | The objective of this review was to assess evaluations of electronic event-based biosurveillance systems. It has no connection to the use of social media in detecting emerging infectious diseases. Accordingly, it doesn't meet the scope of this systematic review. |
| 27 | Gesser-Edelsburg, A., Stolero, N., Mordini, E., Billingsley, M., James, J. J., & Green, M. S. (2015). Emerging infectious disease (EID) communication during the 2009 H1N1 influenza outbreak: literature review (2009-2013) of the methodology used for EID communication analysis. Disaster medicine and public health preparedness, 9(2), 199-206. | The objective of this paper was to conduct a systematic literature review of the methodology used by studies that examined emerging infectious disease communication during the 2009 H1N1 pandemic outbreak through different communication channels, including social media. It has no connection to the use of social media in detecting emerging infectious diseases. Accordingly, it doesn't meet the scope of this systematic review. |
| 28 | Giustini, D., Ali, S. M., Fraser, M., & Boulos, M. N. K. (2018). Effective uses of social media in public health and medicine: a systematic review of systematic reviews. Online journal of public health informatics, 10(2). | The study examined the effective uses of social media in public health and medicine. It is a systematic review of systematic reviews which covers broader topics such as the impact of SM on mental health, benefits from patients and health professional. Indeed, the study includes only one paper selected also in our review (Charles-Smith et al., 2015). |
| 29 | Hamm, M. P., Chisholm, A., Shulhan, J., Milne, A., Scott, S. D., Given, L. M., & Hartling, L. (2013). Social media use among patients and caregivers: a scoping review. BMJ open, 3(5). | The aim of the study was to map the state of the existing literature evaluating the use of social media in patient and caregiver populations. It doesn't concern the use of social media in detecting emerging infectious diseases. Accordingly, it doesn't meet the scope of this systematic review. |
| 30 | Hoang, T., Coletti, P., Melegaro, A., Wallinga, J., Grijalva, C. G., Edmunds, J. W., ... & Hens, N. (2019). A systematic review of social contact surveys to inform transmission models of close-contact infections. Epidemiology (Cambridge, Mass.), 30(5), 723. | Authors undertaked a systematic review of the study design, statistical analyses, and outcomes of the many social contact surveys that have been published. Studies including contacts without physical presence (e.g., phone, internet/social media contacts) were excluded. Accordingly, the paper doesn't meet the scope of our review. |
| 31 | Hudnut-Beumler, J., Po'e, E., & Barkin, S. (2016). The use of social media for health promotion in Hispanic populations: a scoping systematic review. JMIR public health and surveillance, 2(2), e32. | Authors conducted a scoping systematic review of the published literature to capture the ways social media has been used in health interventions aimed at Hispanic populations. It doesn't concern the use of social media in detecting emerging infectious diseases. Accordingly, it doesn't meet the scope of this systematic review. |
| 32 | Hunter, R. F., de la Haye, K., Badham, J., Valente, T., Clarke, M., & Kee, F. (2017). Social network interventions for health behaviour change: a systematic review. The Lancet, 390, S47. | The aim of the study was to investigate the effects of social network interventions for health behaviour change. It doesn't concern the use of social media in detecting emerging infectious diseases. Accordingly, it doesn't meet the scope of this systematic review. |
| 33 | Kazemi, D. M., Borsari, B., Levine, M. J., & Dooley, B. (2017). Systematic review of surveillance by social media platforms for illicit drug use. Journal of Public Health, 39(4), 763-776. | Authors conducted a systematic review of literature focused on the ability of social media to better recognize illicit drug use trends was addressed. It doesn't concern the use of social media in detecting emerging infectious diseases. Accordingly, it doesn't meet the scope of this systematic review. |
| 34 | Kpokiri, E. E., Marley, G., Tang, W., Fongwen, N., Wu, D., Berendes, S., ... & Tucker, J. D. (2020, October). Diagnostic infectious diseases testing outside clinics: a global systematic review and meta-analysis. In Open forum infectious diseases (Vol. 7, No. 10, p. ofaa360). US: Oxford University Press. | The purpose of this study was to examine infectious disease diagnostic testing outside of clinics using a systematic review and meta-analysis. It doesn't concern the use of social media in detecting emerging infectious diseases. Accordingly, it doesn't meet the scope of this systematic review. |
| 35 | Kullar, R., Goff, D. A., Gauthier, T. P., & Smith, T. C. (2020). To tweet or not to tweet—a review of the viral power of twitter for infectious diseases. Current Infectious Disease Reports, 22(6), 1-6. | No systematic review |
| 36 | Laranjo, L., Arguel, A., Neves, A. L., Gallagher, A. M., Kaplan, R., Mortimer, N., ... & Lau, A. Y. (2015). The influence of social networking sites on health behavior change: a systematic review and meta-analysis. Journal of the American Medical Informatics Association, 22(1), 243-256. | The aim of this study was to evaluate the use and effectiveness of interventions using social networking sites (SNSs) to change health behaviors. It doesn't concern the use of social media in detecting emerging infectious diseases. Accordingly, it doesn't meet the scope of this systematic review. |
| 37 | Maher, C. A., Lewis, L. K., Ferrar, K., Marshall, S., De Bourdeaudhuij, I., & Vandelanotte, C. (2014). Are health behavior change interventions that use online social networks effective? A systematic review. Journal of medical Internet research, 16(2), e40. | Authors investigated how online social networks may best be harnessed to achieve health behavior change. It doesn't concern the use of social media in detecting emerging infectious diseases. Accordingly, it doesn't meet the scope of this systematic review. |
| 38 | Mita, G., Ni Mhurchu, C., & Jull, A. (2016). Effectiveness of social media in reducing risk factors for noncommunicable diseases: a systematic review and meta-analysis of randomized controlled trials. Nutrition reviews, 74(4), 237-247. | The primary aim of the current study was to synthesize evidence of the effect of social media use compared with no social media use as part of interventions to reduce risk factors for noncommunicable diseases which include obesity, physical inactivity, and low fruit and vegetable intake. It doesn't meet the scope of this systematic review. |
| 39 | N.Durga1, N., Hanirex, K., Muthukumaravel, A. (2020). A systematic review on big data analysis in the health care technology. Journal of Critical review, 7(19), 3149-3157. | The study doesn't not meet the criteria of a systematic review. |
| 40 | Ni, M. Y., Yang, L., Leung, C. M., Li, N., Yao, X. I., Wang, Y., ... & Liao, Q. (2020). Mental health, risk factors, and social media use during the COVID-19 epidemic and cordon sanitaire among the community and health professionals in Wuhan, China: cross-sectional survey. JMIR mental health, 7(5), e19009. | The aim of this study was to examine risk factors, including the use of social media, for probable anxiety and depression in the community and among health professionals in the epicenter, Wuhan, China. It doesn't meet the scope of this systematic review. |
| 41 | Nuti, S. V., Wayda, B., Ranasinghe, I., Wang, S., Dreyer, R. P., Chen, S. I., & Murugiah, K. (2014). The use of google trends in health care research: a systematic review. PloS one, 9(10), e109583. | Authors performed a systematic review of the health care literature to characterize how researchers use Google Trends. It doesn't concern the use of social media in detecting emerging infectious diseases. Accordingly, it doesn't meet the scope of this systematic review. |
| 42 | Perkins, J. M., Subramanian, S. V., & Christakis, N. A. (2015). Social networks and health: a systematic review of sociocentric network studies in low-and middle-income countries. Social science & medicine, 125, 60-78. | The aim of this study was to summarize existing knowledge about network structure and function in relation to health in low- and middle-income countries. It doesn't meet the scope of this systematic review. |
| 43 | Quinn, E., Hsiao, K. H., Maitland-Scott, I., Gomez, M., Baysari, M. T., Najjar, Z., & Gupta, L. (2021). Web-Based Apps for Responding to Acute Infectious Disease Outbreaks in the Community: Systematic Review. JMIR public health and surveillance, 7(4), e24330. | This review focuses on software apps that collate and analyze communicable disease outbreak data. Social media are not included. Accordingly, it doesn't meet the scope of this systematic review. |
| 44 | Schein, R., Wilson, K., & Keelan, J. E. (2011). Literature review on effectiveness of the use of social media: a report for Peel Public Health. [Region of Peel], Peel Public Health. | Book |
| 45 | Shieh, C., Khan, I., & Umoren, R. (2020). Engagement design in studies on pregnancy and infant health using social media: Systematic review. Preventive Medicine Reports, 19. | This systematic review analyzed participant engagement design in studies using social media and focused on pregnancy and infant health. It doesn't concern the use of social media in detecting emerging infectious diseases. Accordingly, it doesn't meet the scope of this systematic review. |
| 46 | Steele, L., Orefuwa, E., & Dickmann, P. (2016). Drivers of earlier infectious disease outbreak detection: a systematic literature review. International Journal of Infectious Diseases, 53, 15-20. | Authors performed a systematic review of the peer-reviewed literature to identify what evidence exists about factors that influence earlier detection of infectious disease outbreaks. It doesn't include the role of social media in detecting emerging infectious diseases. Accordingly, it doesn't meet the scope of this systematic review. |
| 47 | Suarez-Lledo, V., & Alvarez-Galvez, J. (2021). Prevalence of health misinformation on social media: systematic review. Journal of medical Internet research, 23(1), e17187. | This systematic review aimed to identify the main health misinformation topics and their prevalence on different social media platforms, focusing on methodological quality and the diverse solutions that are being implemented to address this public health concern. It doesn't concern the use of social media in detecting emerging infectious diseases. Accordingly, it doesn't meet the scope of this systematic review. |
| 48 | Swaan, C., van den Broek, A., Kretzschmar, M., & Richardus, J. H. (2018). Timeliness of notification systems for infectious diseases: A systematic literature review. PloS one, 13(6), e0198845. | A systematic literature review was performed to assess outcomes of studies on notification timeliness and to determine which aspects of notification systems are associated with timely notification. It doesn't concern the use of social media in detecting emerging infectious diseases. Accordingly, it doesn't meet the scope of this systematic review. |
| 49 | Taggart, T., Grewe, M. E., Conserve, D. F., Gliwa, C., & Isler, M. R. (2015). Social media and HIV: a systematic review of uses of social media in HIV communication. Journal of medical Internet research, 17(11), e248. | Authors investigated the use of social media as part of human immunodeficiency virus (HIV) prevention and treatment efforts. It doesn't meet the scope of this systematic review. |
| 50 | Tom-Aba, D., Nguku, P. M., Arinze, C. C., & Krause, G. (2018). Assessing the concepts and designs of 58 mobile apps for the management of the 2014-2015 West Africa Ebola outbreak: Systematic review. JMIR public health and surveillance, 4(4), e68. | Authors conducted a systematic review of mHealth tools in the context of the recent Ebola virus disease outbreak to identify the most promising approaches and guide further mHealth developments for infectious disease control. It has no connection to social media. Accordingly, it doesn't meet the scope of this systematic review. |
| 51 | Tursunbayeva, A., Franco, M., & Pagliari, C. (2017). Use of social media for e-Government in the public health sector: A systematic review of published studies. Government Information Quarterly, 34(2), 270-282. | Authors investigated the adoption and use of social media by public health organization, but it doesn't concern the use of social media in detecting emerging infectious diseases. Accordingly, it doesn't meet the scope of this systematic review. |
| 52 | Verelst, F., Willem, L., & Beutels, P. (2016). Behavioural change models for infectious disease transmission: a systematic review (2010–2015). Journal of The Royal Society Interface, 13(125), 20160820. | The main goal of this paper is to systematically review and document how and to which extent behavioural immunity has been explored in infectious disease transmission models. It doesn't concern the use of social media in detecting emerging infectious diseases. Accordingly, it doesn't meet the scope of this systematic review. |
| 53 | Vrbova, L., Stephen, C., Kasman, N., Boehnke, R., Doyle‐Waters, M., Chablitt‐Clark, A., ... & Patrick, D. M. (2010). Systematic review of surveillance systems for emerging zoonoses. Transboundary and emerging diseases, 57(3), 154-161. | This review documented the extent of emerging infectious disease surveillance system evaluation, determined what criteria have been used to evaluate these systems and sought common features of successful systems. It doesn't include social media. Accordingly, it doesn't meet the scope of this systematic review. |
| 54 | Wang, Y., McKee, M., Torbica, A., & Stuckler, D. (2019). Systematic literature review on the spread of health-related misinformation on social media. Social Science & Medicine, 240, 112552. | Authors reported a systematic review of the nature and potential drivers of health-related misinformation. It doesn't concern the use of social media in detecting emerging infectious diseases. Accordingly, it doesn't meet the scope of this systematic review. |
| 55 | Weston, D., Hauck, K., & Amlôt, R. (2018). Infection prevention behaviour and infectious disease modelling: a review of the literature and recommendations for the future. BMC public health, 18(1), 1-16. | This paper presents a large scale scoping review regarding the incorporation of infection prevention behaviour in infectious disease models. The outcomes of this review are contextualised within the psychological literature concerning health behaviour and behaviour change, resulting in a series of key recommendations for the incorporation of human behaviour in future infectious disease models. It doesn't meet the scope of this systematic review. |
| 56 | Zhang, R., & Fu, J. S. (2020). Linking Network Characteristics of Online Social Networks to Individual Health: A Systematic Review of Literature. Health Communication, 1-11. | This paper presents a systematic review of the literature examining how network characteristics of online social networks are linked to individuals' health behavior and/or status. It doesn't concern the use of social media in detecting emerging infectious diseases. Accordingly, it doesn't meet the scope of this systematic review. |
| 57 | Ramamoorthy, T., Karmegam, D., & Mappillairaju, B. (2021). Use of social media data for disease based social network analysis and network modeling: A Systematic Review. Informatics for Health and Social Care, 1-12. | Complete paper not available |
| 58 | Sivaprasad, A., Beevi, N. S., & Manojkumar, T. K. (2020). Dengue and Early Warning Systems: A review based on Social Network Analysis. Procedia Computer Science, 171, 253-262. | Authors reviewed the literature on the development of different early warning systems using the techniques of main path analysis and article clustering. They used Pajek and Gephi which are two popular tools employed for Social Network Analysis. This explains the use in the title of the term "social network". Despite this, it doesn't concern the use of social media in detecting emerging infectious diseases. Accordingly, it doesn't meet the scope of this systematic review. |
| 59 | Tang, L., Bie, B., Park, S. E., & Zhi, D. (2018). Social media and outbreaks of emerging infectious diseases: A systematic review of literature. American journal of infection control, 46(9), 962-972. | Authors conducted a systematic literature review to identify the major approaches and assess the rigors in published research articles on emerging infectious diseases and social media. Studies about outbreak surveillance, which is the line of research that uses user-generated social media data to track and predict EID outbreaks, are not included in this systematic literature review. |
| 60 | Thomas, T. L., Schrock, C., & Friedman, D. B. (2016). Providing health consumers with emergency information: A systematic review of research examining social media use during public crises. Journal of Consumer Health on the Internet, 20(1-2), 19-40. | This systematic review explored risk and crisis communication literature to examine how researchers have evaluated social media use in public crises. The review mainly concerns communication and does not include insights about the use of social media in detecting emerging infectious diseases. |

**SUPPLEMENTARY MATERIAL 2 – AMSTAR 2: a critical appraisal tool for systematic reviews that include randomised or non-randomised studies of healthcare interventions, or both**

| **1. Did the research questions and inclusion criteria for the review include the components of PICO?** | | | | | |
| --- | --- | --- | --- | --- | --- |
| For Yes:   - Population - Intervention - Comparator group - Outcome | | Optional (recommended)  □ Timeframe for follow-up |    | Yes No |  |
| **2. Did the report of the review contain an explicit statement that the review methods were established prior to the conduct of the review and did the report justify any significant deviations from the protocol?** | | | | | |
|  | For Partial Yes:  The authors state that they had a written protocol or guide that included ALL the following:   - review question(s) - a search strategy - inclusion/exclusion criteria - a risk of bias assessment | For Yes:  As for partial yes, plus the protocol should be registered and should also have specified:   - a meta-analysis/synthesis plan, if appropriate, *and* - a plan for investigating causes of heterogeneity - justification for any deviations from the protocol |      | Yes Partial Yes No |  |
| **3. Did the review authors explain their selection of the study designs for inclusion in the review?** | | | | | |
|  | For Yes, the review should satisfy ONE of the following:   - *Explanation for* including only RCTs - OR *Explanation for* including only NRSI - OR *Explanation for* including both RCTs and NRSI | |    | Yes No |  |
| **4. Did the review authors use a comprehensive literature search strategy?** | | | | | |
|  | For Partial Yes (all the following): | For Yes, should also have (all the following):   - searched the reference lists / bibliographies of included studies - searched trial/study registries - included/consulted content experts in the field - where relevant, searched for grey literature - conducted search within 24 months of completion of the review |  |  |  |
|  | - searched at least 2 databases (relevant to research question) - provided key word and/or search strategy - justified publication restrictions |  |      | Yes Partial Yes No |  |
|  | (e.g. language) |  |  |  |  |
|  | **5. Did the review authors perform study selection in duplicate?** | |  | |  |
|  | For Yes, either ONE of the following:   - at least two reviewers independently agreed on selection of eligible studies and achieved consensus on which studies to include - OR two reviewers selected a sample of eligible studies and achieved good agreement (at least 80 percent), with the remainder selected by one reviewer. | |    | Yes No |  |

| **6. Did the review authors perform data extraction in duplicate?** | | | |
| --- | --- | --- | --- |
| For Yes, either ONE of the following:   - at least two reviewers achieved consensus on which data to extract from included studies - OR two reviewers extracted data from a sample of eligible studies and achieved good agreement (at least 80 percent), with the remainder extracted by one reviewer. | | | - Yes - No |
| **7. Did the review authors provide a list of excluded studies and justify the exclusions?** | | | |
|  | For Partial Yes:  □ provided a list of all potentially relevant studies that were read  in full-text form but excluded from the review | For Yes, must also have:  □ Justified the exclusion from the review of each potentially relevant study | - Yes - Partial Yes - No |
| **8. Did the review authors describe the included studies in adequate detail?** | | | |
|  | For Partial Yes (ALL the following):   - described populations - described interventions - described comparators - described outcomes - described research designs | For Yes, should also have ALL the following:   - described population in detail - described intervention in detail (including doses where relevant) - described comparator in detail (including doses where relevant) - described study’s setting - timeframe for follow-up | - Yes - Partial Yes - No |
| **9. Did the review authors use a satisfactory technique for assessing the risk of bias (RoB) in individual studies that were included in the review?** | | | |
|  | **RCTs**  For Partial Yes, must have assessed RoB from   - unconcealed allocation, *and* - lack of blinding of patients and assessors when assessing outcomes (unnecessary for objective outcomes such as all-   cause mortality) | For Yes, must also have assessed RoB from:   - allocation sequence that was not truly random, *and* - selection of the reported result from among multiple measurements or analyses of a specified outcome | - Yes - Partial Yes - No - Includes only NRSI |
|  | **NRSI**  For Partial Yes, must have assessed RoB:   - from confounding, *and* - from selection bias   **10. Did the review authors report o** | For Yes, must also have assessed RoB:   - methods used to ascertain exposures and outcomes, *and* - selection of the reported result from among multiple measurements or analyses of a specified outcome   **n the sources of funding for the studies inc** | - Yes - Partial Yes - No - Includes only RCTs   **luded in the review?** |
|  | For Yes  □ Must have reported on the sources of funding for individual studies included  Yes in the review. Note: Reporting that the reviewers looked for this information  No but it was not reported by study authors also qualifies | | |

| **11. If meta-analysis was performed did the review authors use appropriate methods for statistical combination of results?** | | | |
| --- | --- | --- | --- |
|  | **RCTs**  For Yes:   - The authors justified combining the data in a meta-analysis   - AND they used an appropriate weighted technique to combine study results and adjusted for heterogeneity if present.   - AND investigated the causes of any heterogeneity | - Yes - No - No meta-analysis conducted |  |
|  | **For NRSI**  For Yes:   - The authors justified combining the data in a meta-analysis   - AND they used an appropriate weighted technique to combine study results, adjusting for heterogeneity if present   - AND they statistically combined effect estimates from NRSI that were adjusted for confounding, rather than combining raw data, or justified combining raw data when adjusted effect estimates were not available   - AND they reported separate summary estimates for RCTs and NRSI separately when both were included in the review | - Yes - No - No meta-analysis conducted |  |
| **12. If meta-analysis was performed, did the review authors assess the potential impact of RoB in individual studies on the results of the meta-analysis or other evidence synthesis?** | | | |
|  | For Yes:   - included only low risk of bias RCTs - OR, if the pooled estimate was based on RCTs and/or NRSI at variable RoB, the authors performed analyses to investigate possible impact of RoB on summary estimates of effect. | - Yes - No - No meta-analysis conducted |  |
| **13. Did the review authors account for RoB in individual studies when interpreting/ discussing the results of the review?** | | | |
|  | For Yes:   - included only low risk of bias RCTs - OR, if RCTs with moderate or high RoB, or NRSI were included the review provided a discussion of the likely impact of RoB on the results | - Yes - No |  |
| **14. Did the review authors provide a satisfactory explanation for, and discussion of, any heterogeneity observed in the results of the review?** | | | |
|  | For Yes:   - There was no significant heterogeneity in the results - OR if heterogeneity was present the authors performed an investigation of sources of any heterogeneity in the results and discussed the impact of this on the results of the review | - Yes - No |  |
| **15. If they performed quantitative synthesis did the review authors carry out an adequate investigation of publication bias (small study bias) and discuss its likely impact on the results of the review?** | | | |
|  | For Yes:  □ performed graphical or statistical tests for publication bias and discussed the likelihood and magnitude of impact of publication bias | - Yes - No - No meta-analysis conducted |  |

| **16. Did the review authors report any potential sources of conflict of interest, including any funding they received for conducting the review?** | | | |
| --- | --- | --- | --- |
|  | For Yes:   - The authors reported no competing interests OR - The authors described their funding sources and how they managed potential conflicts of interest | - Yes - No |  |

**To cite this tool:** Shea BJ, Reeves BC, Wells G, Thuku M, Hamel C, Moran J, Moher D, Tugwell P, Welch V, Kristjansson E, Henry DA. AMSTAR 2: a critical appraisal tool for systematic reviews that include randomised or non-randomised studies of healthcare interventions, or both. BMJ. 2017 Sep 21;358:j4008.

| **Reference** | **Total score** |
| --- | --- |
| Charles-Smith et al., 2015 | **10,3** |
| Moonrhead et al., 2013 | **9,8** |
| Pujante-Otalora et al. 2023 | **9,7** |
| Takats et al., 2022 | **9,1** |
| Choi et al., 2016 | **8,775** |
| Barros et al., 2020 | **8,7** |
| Javier Alvarez-Galvez et al., 2021 | **8,6** |
| Sinnenberg et al., 2016 | **8,2** |
| Luan and Law., 2014 | **8,175** |
| Al-Garadi et al., 2016 | **7,775** |
| Phillips et al., 2017 | **7,6** |
| Fung et al., 2016 | **7,575** |
| Gunasekeran et al., 2022 | **6,85** |
| Gianfredi et al., 2018 | **6,775** |
| Carrol et al., 2014 | **6,6** |
| Eckert et al., 2017 | **6,6** |
| Golinelli et al., 2020 | **6,575** |
| Corsi et al., 2020 | **6,45** |
| Chen and Wang, 2021 | **6,275** |
| Tsao et al., 2021 | **6,2** |
| Bernardo et al., 2013 | **6,175** |
| Gupta and Katarya, 2020 | **6,075** |
| Díaz-Campo et al., 2023 | **6,075** |
| Guy et al., 2011 | **5,975** |
| Hagg et al., 2018 | **5,975** |
| Velasco et al., 2014 | **5,975** |
| Shoaei and Dastani, 2020 | **5,575** |
| Khan et al., 2022 | **5,575** |
| O'Shea, 2017 | **5,475** |
| Pilipiec et al., 2023 | **5,3** |
| Agrawal and Gupta, 2020 | **4,775** |
| de araujo et al., 2018 | **3,075** |

| **Reference** | **1. Did the research questions and inclusion criteria for the review include the components of PICO?** | |
| --- | --- | --- |
|  | **Yes** | **No** |
| Charles-Smith et al., 2015 |  | 0 |
| Moonrhead et al., 2013 |  | 0 |
| Pujante-Otalora et al. 2023 | 1 |  |
| Takats et al., 2022 |  | 0 |
| Choi et al., 2016 |  | 0 |
| Barros et al., 2020 | 1 | 0 |
| Javier Alvarez-Galvez et al., 2021 |  | 0 |
| Sinnenberg et al., 2016 |  | 0 |
| Luan and Law., 2014 |  | 0 |
| Al-Garadi et al., 2016 |  | 0 |
| Phillips et al., 2017 |  | 0 |
| Fung et al., 2016 |  | 0 |
| Gunasekeran et al., 2022 |  | 0 |
| Gianfredi et al., 2018 |  | 0 |
| Carrol et al., 2014 |  | 0 |
| Eckert et al., 2017 |  | 0 |
| Golinelli et al., 2020 |  | 0 |
| Corsi et al., 2020 |  | 0 |
| Chen and Wang, 2021 |  | 0 |
| Tsao et al., 2021 |  | 0 |
| Bernardo et al., 2013 |  | 0 |
| Gupta and Katarya, 2020 |  | 0 |
| Díaz-Campo et al., 2023 |  | 0 |
| Guy et al., 2011 |  | 0 |
| Hagg et al., 2018 |  | 0 |
| Velasco et al., 2014 |  | 0 |
| Shoaei and Dastani, 2020 |  | 0 |
| Khan et al., 2022 |  | 0 |
| O'Shea, 2017 |  | 0 |
| Pilipiec et al., 2023 |  | 0 |
| Agrawal and Gupta, 2020 |  | 0 |
| de araujo et al., 2018 |  | 0 |

| **Reference** | **2. Did the report of the review contain an explicit statement that the review methods were established prior to the conduct of the review and did the report justify any significant deviations from the protocol?** | | | | | | | |
| --- | --- | --- | --- | --- | --- | --- | --- | --- |
|  | **Yes (As for partial yes, plus the protocol should be registered and should also have specified:)** | | | **Partial Yes (The authors state that they had a written protocol or guide that included ALL the following:)** | | | | **No** |
|  | **a meta- analysis/synthesis plan, if appropriate, and (0,34)** | **a plan for investigating causes of heterogeneity (0,34)** | **justification for any deviations from the protocol (0,34)** | **review question(s) (0,125)** | **a search strategy (0,125)** | **inclusion/exclusion criteria (0,125)** | **a risk of bias assessment (0,125)** |  |
| Charles-Smith et al., 2015 | 0 | 0 | 0 | 0,125 | 0,125 | 0,125 | 0,125 |  |
| Moonrhead et al., 2013 | 0 | 0 | 0 | 0,125 | 0,125 | 0,125 | 0,125 |  |
| Pujante-Otalora et al. 2023 | 0 | 0 | 0 | 0,125 | 0,125 | 0,125 | 0,125 |  |
| Takats et al., 2022 | 0 | 0 | 0 | 0,125 | 0,125 | 0,125 | 0,125 |  |
| Choi et al., 2016 | 0 | 0 | 0 | 0,125 | 0,125 | 0,125 | 0 |  |
| Barros et al., 2020 | 0 | 0 | 0 | 0,125 | 0,125 | 0,125 | 0,125 |  |
| Javier Alvarez-Galvez et al., 2021 | 0 | 0 | 0 | 0,125 | 0,125 | 0,125 | 0,125 |  |
| Sinnenberg et al., 2016 | 0 | 0 | 0 | 0,125 | 0,125 | 0,125 | 0,125 |  |
| Luan and Law., 2014 | 0 | 0 | 0 | 0,125 | 0,125 | 0,125 | 0 |  |
| Al-Garadi et al., 2016 | 0 | 0 | 0 | 0,125 | 0,125 | 0,125 | 0 |  |
| Phillips et al., 2017 | 0 | 0 | 0 | 0,125 | 0,125 | 0,125 | 0,125 |  |
| Fung et al., 2016 | 0 | 0 | 0 | 0,125 | 0,125 | 0,125 | 0 |  |
| Gunasekeran et al., 2022 | 0 | 0 | 0 | 0,125 | 0,125 | 0 | 0 |  |
| Gianfredi et al., 2018 | 0 | 0 | 0 | 0,125 | 0,125 | 0,125 | 0 |  |
| Carrol et al., 2014 | 0 | 0 | 0 | 0,125 | 0,125 | 0,125 | 0,125 |  |
| Eckert et al., 2017 | 0 | 0 | 0 | 0,125 | 0,125 | 0,125 | 0,125 |  |
| Golinelli et al., 2020 | 0 | 0 | 0 | 0,125 | 0,125 | 0,125 | 0 |  |
| Corsi et al., 2020 | 0 | 0 | 0 | 0,125 | 0,125 | 0 | 0 |  |
| Chen and Wang, 2021 | 0 | 0 | 0 | 0,125 | 0,125 | 0,125 | 0 |  |
| Tsao et al., 2021 | 0 | 0 | 0 | 0,125 | 0,125 | 0,125 | 0,125 |  |
| Bernardo et al., 2013 | 0 | 0 | 0 | 0,125 | 0,125 | 0,125 | 0 |  |
| Gupta and Katarya, 2020 | 0 | 0 | 0 | 0,125 | 0,125 | 0,125 | 0 |  |
| Díaz-Campo et al., 2023 | 0 | 0 | 0 | 0,125 | 0,125 | 0,125 | 0 |  |
| Guy et al., 2011 | 0 | 0 | 0 | 0,125 | 0,125 | 0,125 | 0 |  |
| Hagg et al., 2018 | 0 | 0 | 0 | 0,125 | 0,125 | 0,125 | 0 |  |
| Velasco et., 2014 | 0 | 0 | 0 | 0,125 | 0,125 | 0,125 | 0 |  |
| Shoaei and Dastani, 2020 | 0 | 0 | 0 | 0,125 | 0,125 | 0,125 | 0 |  |
| Khan et al., 2022 | 0 | 0 | 0 | 0,125 | 0,125 | 0 | 0,125 |  |
| O'Shea, 2017 | 0 | 0 | 0 | 0,125 | 0,125 | 0,125 | 0 |  |
| Pilipiec et al., 2023 | 0 | 0 | 0 | 0,125 | 0,125 | 0,125 | 0,125 |  |
| Agrawal and Gupta, 2020 | 0 | 0 | 0 | 0,125 | 0,125 | 0,125 | 0 |  |
| de araujo et al., 2018 | 0 | 0 | 0 | 0,125 | 0,125 | 0,125 | 0 |  |

| **Reference** | **3. Did the review authors explain their selection of the study designs for inclusion in the review?** | |
| --- | --- | --- |
|  | **YES** | **NO** |
| Charles-Smith et al., 2015 | 1 |  |
| Moonrhead et al., 2013 | 1 |  |
| Pujante-Otalora et al. 2023 | 1 |  |
| Takats et al., 2022 | 1 |  |
| Choi et al., 2016 | 1 |  |
| Barros et al., 2020 | 1 |  |
| Javier Alvarez-Galvez et al., 2021 | 1 |  |
| Sinnenberg et al., 2016 | 1 |  |
| Luan and Law., 2014 | 1 |  |
| Al-Garadi et al., 2016 | 1 |  |
| Phillips et al., 2017 | 1 |  |
| Fung et al., 2016 | 1 |  |
| Gunasekeran et al., 2022 | 1 |  |
| Gianfredi et al., 2018 | 1 |  |
| Carrol et al., 2014 | 1 |  |
| Eckert et al., 2017 | 1 |  |
| Golinelli et al., 2020 | 1 |  |
| Corsi et al., 2020 | 1 |  |
| Chen and Wang, 2021 | 1 |  |
| Tsao et al., 2021 | 1 |  |
| Bernardo et al., 2013 | 1 |  |
| Gupta and Katarya, 2020 | 1 |  |
| Díaz-Campo et al., 2023 | 1 |  |
| Guy et al., 2011 | 1 |  |
| Hagg et al., 2018 | 1 |  |
| Velasco et al., 2014 | 1 |  |
| Shoaei and Dastani, 2020 | 1 |  |
| Khan et al., 2022 | 1 |  |
| O'Shea, 2017 | 1 |  |
| Pilipiec et al., 2023 | 1 |  |
| Agrawal and Gupta, 2020 | 1 |  |
| de araujo et al., 2018 | 1 |  |

| **Reference** | **4. Did the review authors use a comprehensive literature search strategy?** | | | | | | | | |
| --- | --- | --- | --- | --- | --- | --- | --- | --- | --- |
|  | **Yes ((For Yes, should also have (all the following) :)** | | | | | **Partial Yes ((For Partial Yes (all the following) :)** | | | **No** |
|  | **searched the reference lists/bibliographies of included studies (0,2)** | **searched trial/stud y registries (0,2)** | **included/consulte d content experts in the field (0,2)** | **where relevant, searched for grey literature (0,2)** | **conducted search within 24 months of completio n of the review (0,2)** | **searched at least 2 database s (relevant to research question) (0,16..7)** | **provide d key word and/or search strategy (0,16..7)** | **justified publicatio n restriction s (eg, language) (0,16..7)** |  |
| Charles-Smith et al., 2015 | 0,2 | 0 | 0,2 | 0,2 | 0,2 | 0,16..7 | 0,16..7 | 0,16..7 |  |
| Moonrhead et al., 2013 | 0,2 | 0,2 | 0 | 0,2 | 0,2 | 0,16..7 | 0,16..7 | 0,16..7 |  |
| Pujante-Otalora et al. 2023 | 0 | 0 | 0 | 0 | 0,2 | 0,16..7 | 0,16..7 | 0 |  |
| Takats et al., 2022 | 0,2 | 0 | 0,2 | 0 | 0,2 | 0,16..7 | 0,16..7 | 0 |  |
| Choi et al., 2016 | 1 | 0 | 0,2 | 0 | 0,2 | 0,16..7 | 0,16..7 | 0,16..7 |  |
| Barros et al., 2020 | 0 | 0 | 0 | 0 | 0,2 | 0,16..7 | 0,16..7 | 0,16..7 |  |
| Javier Alvarez-Galvez et al., 2021 | 0,2 | 0 | 0,2 | 0 | 0,2 | 0,16..7 | 0,16..7 | 0,16..7 |  |
| Sinnenberg et al., 2016 | 0 | 0 | 0 | 0 | 0,2 | 0,16..7 | 0,16..7 | 0,16..7 |  |
| Luan and Law., 2014 | 0,2 |  | 0,2 | 0,2 | 0,2 | 0,16..7 | 0,16..7 | 0,16..7 |  |
| Al-Garadi et al., 2016 | 0,2 | 0 | 0 | 0 | 0,2 | 0,16..7 | 0,16..7 | 0 |  |
| Phillips et al., 2017 | 0,2 | 0 | 0 | 0,2 | 0,2 | 0,16..7 | 0,16..7 | 0,16..7 |  |
| Fung et al., 2016 | 0 | 0 | 0 | 0 | 0,2 | 0,16..7 | 0,16..7 | 0,16..7 |  |
| Gunasekeran et al., 2022 | 0,2 | 0 | 0,2 | 0 | 0,2 | 0,16..7 | 0,16..7 | 0 |  |
| Gianfredi et al., 2018 | 0,2 | 0 | 0 | 0 | 0,2 | 0,16..7 | 0,16..7 | 0,16..7 |  |
| Carrol et al., 2014 | 0,2 | 0 | 0 | 0,2 | 0,2 | 0,16..7 | 0,16..7 | 0,16..7 |  |
| Eckert et al., 2017 | 0 | 0,2 | 0 | 0,2 | 0,2 | 0,16..7 | 0,16..7 | 0,16..7 |  |
| Golinelli et al., 2020 | 0 | 0 | 0 | 0 | 0,2 | 0,16..7 | 0,16..7 | 0,16..7 |  |
| Corsi et al., 2020 | 0 | 0 | 0 | 0 | 0,2 | 0,16..7 | 0,16..7 | 0,16..7 |  |
| Chen and Wang, 2021 | 0 | 0,2 | 0 | 0 | 0,2 | 0,16..7 | 0,16..7 | 0,16..7 |  |
| Tsao et al., 2021 | 0 | 0 | 0 | 0 | 0,2 | 0,16..7 | 0,16..7 | 0,16..7 |  |
| Bernardo et al., 2013 | 0,2 | 0 | 0,2 | 0,2 | 0,2 | 0 | 0,16..7 | 0,16..7 |  |
| Gupta and Katarya, 2020 | 0 | 0 | 0 | 0 | 0,2 | 0,16..7 | 0,16..7 | 0,16..7 |  |
| Díaz-Campo et al., 2023 | 0 | 0 | 0 | 0 | 0,2 | 0,16..7 | 0,16..7 | 0 |  |
| Guy et al., 2011 | 0,2 | 0 | 0 | 0,2 | 0,2 | 0,16..7 | 0,16..7 | 0,16..7 |  |
| Hagg et al., 2018 | 0,2 | 0 | 0 | 0,2 | 0,2 | 0,16..7 | 0,16..7 | 0,16..7 |  |
| Velasco et al., 2014 | 0,2 | 0 | 0,2 | 0 | 0,2 | 0,16..7 | 0,16..7 | 0,16..7 |  |
| Shoaei and Dastani, 2020 | 0 | 0 | 0 | 0 | 0,2 | 0,16..7 | 0,16..7 | 0,16..7 |  |
| Khan et al., 2022 | 0 | 0 | 0 | 0 | 0,2 | 0,16..7 | 0,16..7 | 0 |  |
| O'Shea, 2017 | 0,2 | 0 | 0 | 0,2 | 0,2 | 0,16..7 | 0,16..7 | 0,16..7 |  |
| Pilipiec et al., 2023 | 0,2 | 0,2 | 0 | 0,2 | 0,2 | 0,16..7 | 0,16..7 | 0,16..7 |  |
| Agrawal and Gupta, 2020 | 0,2 | 0 | 0 | 0 | 0,2 | 0,16..7 | 0,16..7 | 0,16..7 |  |
| de araujo et al., 2018 | 0 | 0 | 0 | 0 | 0,2 | 0,16..7 | 0,16..7 | 0,16..7 |  |

| **Reference** | **5. Did the review authors perform study selection in duplicate?** | | **6. Did the review authors perform data extraction in duplicate?** | | **7. Did the review authors provide a list of excluded studies and justify the exclusions?** | | | **8. Did the review authors describe the included studies in adequate detail?** | | |
| --- | --- | --- | --- | --- | --- | --- | --- | --- | --- | --- |
|  | **Yes** | **No** | **Yes** | **No** | **Yes** | **Partial Yes** | **No** | **Yes** | **Partial Yes** | **No** |
| Charles-Smith et al., 2015 | 1 |  | 1 |  |  |  | 0 | 1 |  |  |
| Moonrhead et al., 2013 | 1 |  | 1 |  | 1 |  |  | 1 |  |  |
| Pujante-Otalora et al. 2023 | 1 |  | 1 |  |  |  | 0 | 1 |  |  |
| Takats et al., 2022 | 1 |  | 1 |  |  |  | 0 | 1 |  |  |
| Choi et al., 2016 | 1 |  | 1 |  |  |  | 0 | 1 |  |  |
| Barros et al., 2020 |  | 0 | 1 |  |  |  | 0 | 1 |  |  |
| Javier Alvarez-Galvez et al., 2021 | 1 |  | 1 |  |  |  | 0 | 1 |  |  |
| Sinnenberg et al., 2016 | 1 |  | 1 |  |  |  | 0 | 1 |  |  |
| Luan and Law., 2014 | 1 |  | 1 |  | 1 |  |  | 1 |  |  |
| Al-Garadi et al., 2016 | 1 |  | 1 |  |  |  | 0 | 1 |  |  |
| Phillips et al., 2017 | 1 |  | 1 |  |  |  | 0 | 1 |  |  |
| Fung et al., 2016 | 1 |  | 1 |  | 1 |  |  | 1 |  |  |
| Gunasekeran et al., 2022 | 1 |  | 1 |  |  |  | 0 | 1 |  |  |
| Gianfredi et al., 2018 | 1 |  | 1 |  |  |  | 0 | 1 |  |  |
| Carrol et al., 2014 | 0 |  | 0 |  |  |  | 0 | 1 |  |  |
| Eckert et al., 2017 |  | 0 |  | 0 |  |  | 0 | 1 |  |  |
| Golinelli et al., 2020 | 1 | 0 | 1 | 0 |  |  | 0 | 1 |  |  |
| Corsi et al., 2020 | 1 |  | 1 |  |  |  | 0 | 1 |  |  |
| Chen and Wang, 2021 | 1 |  | 1 |  |  |  | 0 | 1 |  |  |
| Tsao et al., 2021 | 1 |  | 1 |  |  |  | 0 | 1 |  |  |
| Bernardo et al., 2013 | 1 |  |  | 0 |  |  | 0 | 1 |  |  |
| Gupta and Katarya, 2020 | 1 |  | 1 |  |  |  | 0 | 1 |  |  |
| Díaz-Campo et al., 2023 | 1 |  | 1 |  |  | 0 |  | 1 |  |  |
| Guy et al., 2011 | 1 |  | 1 |  |  |  | 0 | 1 |  |  |
| Hagg et al., 2018 | 0 |  | 1 |  |  |  | 0 | 1 |  |  |
| Velasco et al., 2014 | 1 |  | 1 |  |  |  | 0 | 1 |  |  |
| Shoaei and Dastani, 2020 | 1 |  | 1 |  |  |  | 0 | 1 |  |  |
| Khan et al., 2022 | 1 |  | 1 |  |  |  | 0 | 1 |  |  |
| O'Shea, 2017 |  | 0 | 1 |  |  |  | 0 | 1 |  |  |
| Pilipiec et al., 2023 |  | 0 |  | 0 |  |  | 0 | 1 |  |  |
| Agrawal and Gupta, 2020 |  | 0 |  | 0 |  |  | 0 | 1 |  |  |
| de araujo et al., 2018 |  | 0 |  | 0 | 1 |  |  |  | 0,5 |  |

| **Reference** | **9. Did the review authors use a satisfactory technique for assessing the risk of bias (RoB) in individual studies that were included in the review?** | | | **10. Did the review authors report on the sources of funding for the studies included in the review?** | | **11. If meta-analysis was performed did the review authors use appropriate methods for statistical combination of results?** | | | **12. If meta-analysis was performed, did the review authors assess the potential impact of RoB in individual studies on the results of the meta-analysis or other evidence synthesis?** | | |
| --- | --- | --- | --- | --- | --- | --- | --- | --- | --- | --- | --- |
|  | **Yes** | **Partial Yes** | **No** | **Yes** | **No** | **Yes** | **No** | **No meta-analysis conducted** | **Yes** | **No** | **No meta-analysis conducted** |
| Charles-Smith et al., 2015 | 1 |  |  | 1 |  |  |  | 0 |  |  | 0 |
| Moonrhead et al., 2013 | 1 |  |  |  | 0 |  |  | 0 |  |  | 0 |
| Pujante-Otalora et al. 2023 | 1 |  |  |  | 0 |  |  | 0 |  |  | 0 |
| Takats et al., 2022 | 1 |  |  |  | 0 |  |  | 0 |  |  | 0 |
| Choi et al., 2016 | 1 |  |  |  | 0 |  |  | 0 |  |  | 0 |
| Barros et al., 2020 | 1 |  |  |  | 0 |  |  | 0 |  |  | 0 |
| Javier Alvarez-Galvez et al., 2021 |  | 0,5 |  |  | 0 |  |  | 0 |  |  | 0 |
| Sinnenberg et al., 2016 |  |  | 0 | 1 |  |  |  | 0 |  |  | 0 |
| Luan and Law., 2014 |  |  | 0 |  | 0 |  |  | 0 |  |  | 0 |
| Al-Garadi et al., 2016 | 1 |  |  |  | 0 |  |  | 0 |  |  | 0 |
| Phillips et al., 2017 |  | 0,5 |  |  | 0 |  |  | 0 |  |  | 0 |
| Fung et al., 2016 |  |  | 0 |  | 0 |  |  | 0 |  |  | 0 |
| Gunasekeran et al., 2022 |  |  | 0 |  | 0 |  |  | 0 |  |  | 0 |
| Gianfredi et al., 2018 |  |  | 0 |  | 0 |  |  | 0 |  |  | 0 |
| Carrol et al., 2014 | 1 |  |  |  | 0 |  |  | 0 |  |  | 0 |
| Eckert et al., 2017 | 1 |  |  |  | 0 |  |  | 0 |  |  | 0 |
| Golinelli et al., 2020 |  |  | 0 |  | 0 |  |  | 0 |  |  | 0 |
| Corsi et al., 2020 |  |  | 0 |  | 0 |  |  | 0 |  |  | 0 |
| Chen and Wang, 2021 |  |  | 0 |  | 0 |  |  | 0 |  |  | 0 |
| Tsao et al., 2021 |  |  | 0 |  | 0 |  |  | 0 |  |  | 0 |
| Bernardo et al., 2013 |  |  | 0 |  | 0 |  |  | 0 |  |  | 0 |
| Gupta and Katarya, 2020 |  |  | 0 |  | 0 |  |  | 0 |  |  | 0 |
| Díaz-Campo et al., 2023 |  |  | 0 |  | 0 |  |  | 0 |  |  | 0 |
| Guy et al., 2011 |  |  | 0 |  | 0 |  |  | 0 |  |  | 0 |
| Hagg et al., 2018 |  |  | 0 |  | 0 |  |  | 0 |  |  | 0 |
| Velasco et al., 2014 |  |  | 0 |  | 0 |  |  | 0 |  |  | 0 |
| Shoaei and Dastani, 2020 |  |  | 0 |  | 0 |  |  | 0 |  |  | 0 |
| Khan et al., 2022 |  |  | 0 |  | 0 |  |  | 0 |  |  | 0 |
| O'Shea, 2017 |  |  | 0 |  | 0 |  |  | 0 |  |  | 0 |
| Pilipiec et al., 2023 |  |  | 0 |  | 0 |  |  | 0 |  |  | 0 |
| Agrawal and Gupta, 2020 |  |  | 0 |  | 0 |  |  | 0 |  |  | 0 |
| de araujo et al., 2018 |  |  | 0 |  | 0 |  |  | 0 |  |  | 0 |

| **Reference** | **13. Did the review authors account for RoB in individual studies when interpreting/discussing the results of the review?** | | **14. Did the review authors provide a satisfactory explanation for, and discussion of, any heterogeneity observed in the results of the review?** | | **15. If they performed quantitative synthesis did the review authors carry out an adequate investigation of publication bias (small study bias) and discuss its likely impact on the results of the review?** | | |
| --- | --- | --- | --- | --- | --- | --- | --- |
|  | **Yes** | **No** | **Yes** | **No** | **Yes** | **No** | **No meta-analysis conducted** |
| Charles-Smith et al., 2015 | 1 |  | 1 |  |  |  | 0 |
| Moonrhead et al., 2013 | 1 |  | 1 |  |  |  | 0 |
| Pujante-Otalora et al. 2023 | 1 |  | 1 |  |  |  | 0 |
| Takats et al., 2022 | 1 |  | 1 |  |  |  | 0 |
| Choi et al., 2016 |  | 0 | 1 |  |  |  | 0 |
| Barros et al., 2020 | 1 |  | 1 |  |  |  | 0 |
| Javier Alvarez-Galvez et al., 2021 | 1 |  | 1 |  |  |  | 0 |
| Sinnenberg et al., 2016 | 1 |  | 1 |  |  |  | 0 |
| Luan and Law., 2014 |  | 0 | 1 |  |  |  | 0 |
| Al-Garadi et al., 2016 |  | 0 | 1 |  |  |  | 0 |
| Phillips et al., 2017 | 1 |  | 1 |  |  |  | 0 |
| Fung et al., 2016 |  | 0 | 1 |  |  |  | 0 |
| Gunasekeran et al., 2022 |  | 0 | 1 |  |  |  | 0 |
| Gianfredi et al., 2018 |  | 0 | 1 |  |  |  | 0 |
| Carrol et al., 2014 | 1 |  | 1 |  |  |  | 0 |
| Eckert et al., 2017 | 1 |  | 1 |  |  |  | 0 |
| Golinelli et al., 2020 |  | 0 | 1 |  |  |  | 0 |
| Corsi et al., 2020 |  | 0 | 1 |  |  |  | 0 |
| Chen and Wang, 2021 |  | 0 | 1 |  |  |  | 0 |
| Tsao et al., 2021 |  | 0 | 1 |  |  |  | 0 |
| Bernardo et al., 2013 |  | 0 | 1 |  |  |  | 0 |
| Gupta and Katarya, 2020 |  | 0 | 1 |  |  |  | 0 |
| Díaz-Campo et al., 2023 |  | 0 | 1 |  |  |  | 0 |
| Guy et al., 2011 |  | 0 | 1 |  |  |  | 0 |
| Hagg et al., 2018 |  | 0 | 1 |  |  |  | 0 |
| Velasco et al., 2014 |  | 0 | 1 |  |  |  | 0 |
| Shoaei and Dastani, 2020 |  | 0 | 1 |  |  |  | 0 |
| Khan et al., 2022 |  | 0 | 1 |  |  |  | 0 |
| O'Shea, 2017 |  | 0 | 1 |  |  |  | 0 |
| Pilipiec et al., 2023 |  | 0 | 1 |  |  |  | 0 |
| Agrawal and Gupta, 2020 |  | 0 | 1 |  |  |  | 0 |
| de araujo et al., 2018 |  | 0 |  | 0 |  |  | 0 |

| **Reference** | **16. Did the review authors report any potential sources of conflict of interest, including any funding they received for conducting the review?** | | | |
| --- | --- | --- | --- | --- |
|  | **Yes** | **Partial Yes** | | **No** |
|  |  | **Funding** | **Conflict of interest** |  |
| Charles-Smith et al., 2015 | 1 |  |  |  |
| Moonrhead et al., 2013 |  | 0 | 0,5 |  |
| Pujante-Otalora et al. 2023 | 1 |  |  |  |
| Takats et al., 2022 | 1 |  |  |  |
| Choi et al., 2016 | 1 |  |  |  |
| Barros et al., 2020 | 1 |  |  |  |
| Javier Alvarez-Galvez et al., 2021 | 1 |  |  |  |
| Sinnenberg et al., 2016 |  | 0,5 | 0 |  |
| Luan and Law., 2014 | 1 |  |  |  |
| Al-Garadi et al., 2016 | 1 |  |  |  |
| Phillips et al., 2017 |  |  |  | 0 |
| Fung et al., 2016 | 1 |  |  |  |
| Gunasekeran et al., 2022 |  |  | 0,5 |  |
| Gianfredi et al., 2018 | 1 |  |  |  |
| Carrol et al., 2014 |  | 0,5 | 0 |  |
| Eckert et al., 2017 |  | 0,5 | 0 |  |
| Golinelli et al., 2020 | 1 |  |  |  |
| Corsi et al., 2020 | 1 |  |  |  |
| Chen and Wang, 2021 |  | 0 | 0,5 |  |
| Tsao et al., 2021 |  | 0 | 0,5 |  |
| Bernardo et al., 2013 | 1 |  |  |  |
| Gupta and Katarya, 2020 |  | 0 | 0,5 |  |
| Díaz-Campo et al., 2023 | 1 |  |  |  |
| Guy et al., 2011 |  |  |  | 0 |
| Hagg et al., 2018 | 1 |  |  |  |
| Velasco et al., 2014 |  |  |  | 0 |
| Shoaei and Dastani, 2020 |  |  |  | 0 |
| Khan et al., 2022 |  |  |  | 0 |
| O'Shea, 2017 |  | 0 | 0,5 |  |
| Pilipiec et al., 2023 | 1 |  |  |  |
| Agrawal and Gupta, 2020 | 1 |  |  |  |
| de araujo et al., 2018 |  |  |  | 0 |
